# Systematic evaluation of library preparation methods and sequencing platforms for high-throughput whole genome bisulfite sequencing

**DOI:** 10.1038/s41598-019-46875-5

**Published:** 2019-07-17

**Authors:** Li Zhou, Hong Kiat Ng, Daniela I. Drautz-Moses, Stephan C. Schuster, Stephan Beck, Changhoon Kim, John Campbell Chambers, Marie Loh

**Affiliations:** 1 Lee Kong Chian School of Medicine, Nanyang Technological University, Singapore, 308232 Singapore; 2 Singapore Centre for Environmental Life Sciences Engineering, Nanyang Technological University, Singapore, 637551 Singapore; 3 University College London Cancer Institute, London, WC1E 6BT United Kingdom; 4 Macrogen, Inc., Seoul, 08511 Republic of Korea; 5 Department of Epidemiology and Biostatistics, Imperial College London, London, W2 1PG United Kingdom; 6 Department of Cardiology, Ealing Hospital, London North West Healthcare NHS Trust, Southall, UB1 3HW United Kingdom; 7 Imperial College Healthcare NHS Trust, London, W12 0HS United Kingdom; 8 Translational Laboratory in Genetic Medicine, Agency for Science, Technology and Research (A*STAR), Singapore, 138648 Singapore

**Keywords:** Biotechnology, Genomics, Sequencing

## Abstract

Whole genome bisulfite sequencing (WGBS), with its ability to interrogate methylation status at single CpG site resolution epigenome-wide, is a powerful technique for use in molecular experiments. Here, we aim to advance strategies for accurate and efficient WGBS for application in future large-scale epidemiological studies. We systematically compared the performance of three WGBS library preparation methods with low DNA input requirement (Swift Biosciences Accel-NGS, Illumina TruSeq and QIAGEN QIAseq) on two state-of-the-art sequencing platforms (Illumina NovaSeq and HiSeq X), and also assessed concordance between data generated by WGBS and methylation arrays. Swift achieved the highest proportion of CpG sites assayed and effective coverage at 26x (P < 0.001). TruSeq suffered from the highest proportion of PCR duplicates, while QIAseq failed to deliver across all quality metrics. There was little difference in performance between NovaSeq and HiSeq X, with the exception of higher read duplication rate on the NovaSeq (P < 0.05), likely attributable to the higher cluster densities on its flow cells. Systematic biases exist between WGBS and methylation arrays, with lower precision observed for WGBS across the range of depths investigated. To achieve a level of precision broadly comparable to the methylation array, a minimum coverage of 100x is recommended.

## Introduction

Epigenetic modifications contribute to gene regulation in a heritable fashion without affecting the underlying genomic sequences. DNA methylation, one of the most well-characterized epigenetic modifications, involves the conversion of cytosine to 5-methylcytosine via the covalent transfer of a methyl group to the fifth carbon position of cytosine to form 5-methylcytosine. This heritable epigenetic mark remains stable during cell division and acts as a form of cellular memory, regulating various cellular activities such as transcription and chromosomal stability, and plays a crucial role in embryonic development, genomic imprinting and X-chromosome inactivation. In particular, aberrant DNA methylation has been found to be associated with various complex human diseases such as cancer, autoimmune diseases, metabolic disorders, type-2 diabetes and obesity^1–5^.

Recent advancements in technology has now rendered it possible to investigate the link between DNA methylation and various human phenotypes in a high-throughput fashion. Over the last decade, more than 300 epigenome-wide association studies (EWAS) have been published, with more than half in the last two years alone. This rapid increase in the publication of EWAS studies not only reflect the advancement in technology for measurement of methylation, but is also likely influenced by the success of initial EWASes. Notably, almost all of these studies (~99%) were exclusively performed on methylation arrays. Although the development of methylation arrays has made DNA methylation analyses much more affordable, these arrays remain largely inefficient, covering less than 3% of the CpG sites in the human genome, even on the latest Illumina MethylationEPIC Beadchip^6^. In addition, as the contents of these arrays were determined by expert panels, the selected CpG sites present a biased representation of the genome.

In contrast, whole genome bisulfite sequencing (WGBS) is able to reveal methylation status at each cytosine across the whole genome, with approximately 95% of all CpG sites in the human genome assessable via WGBS^6^. Since the establishment of the first human genome-wide, single-base-resolution DNA methylation map in 2009 by WGBS^7^, the technique has been utilized in the investigation of the relationship between DNA methylation loci and human phenotypes in both basic and clinical research^8^, albeit in small scale studies or at very low coverage. The widespread utilization of WGBS, particularly in EWAS studies, has been hindered primarily by its prohibitively high cost and the large DNA input required, compounded by the extensive computational power and expertise necessary for its accurate interpretation.

With maturation of the next generation sequencing (NGS) technology and improvements in library preparation methods^9^, both the cost of WGBS and the DNA input amount necessary has greatly reduced, rendering this technology increasingly affordable for usage in EWAS and other studies. It is therefore now timely to establish clear recommendations for the generation of high quality WGBS data in view of the repertoire of library preparation methods and sequencing platforms available, which ultimately impacts upon the quantification and interpretation of methylation data.

As previous studies have focused on comparing established pre- and post-bisulfite WGBS library methods as well as short-read sequencing using older HiSeq platforms and high PhiX spike-in^10–12^, there remains a wide knowledge gap for comparison of library preparation method performances with respect to WGBS experiments performed on the latest generation of Illumina sequencing platforms, along with lower PhiX spike-in. The International Human Epigenome Consortium (IHEC) requires single replicate 30x coverage for reference methylomes, and depending on analysis features, recommends multiple replicates based on saturation and recovery analysis^13,14^. Although the National Institutes of Health (NIH) Roadmap Epigenomics Project recommends the use of two replicates with a combined total coverage of 30x^15^, the exact rationale for this guideline is unclear.

Therefore, in this study, we aim to (i) systematically compare the performance of three WGBS library preparation methods with low DNA input requirement across two state-of-the-art sequencing platforms in terms of data quality, (ii) assess the agreement between data generated by WGBS versus that from methylation arrays, and finally, (iii) provide a data-driven recommendation for genomic coverage for future WGBS studies.

## Results

### Comparison of library preparation methods

To systematically compare the performance across different library preparation methods, we selected three kits, namely Swift Biosciences Accel-NGS Methyl-Seq DNA Library kit (Swift thereafter)^16^, Illumina TruSeq DNA Methylation kit (previously known as EpiGnome Methyl-Seq kit, TruSeq thereafter)^17^ and QIAGEN QIAseq Methyl Library kit (QIAseq thereafter) in view of their low DNA input requirement (Table 1). All libraries were sequenced on the HiSeq X platform at 150 bp paired end (PE). DNA samples were previously extracted from whole blood for Samples 1–4 and from isolated white blood cell subsets for Samples 5–8 (CD4^+^ T cells: Samples 5 and 8; Neutrophils: Samples 6 and 7). Library preparation and sequencing protocols were carried out by two independent sequencing providers according to their respective best practice.

**Table 1:** Experimental setup for comparison of library preparation methods.

Provider	A			B		
Samples	Samples 1–4			Samples 5–8		
Sample Type	Whole Blood			Isolated cell subtypes		
Library preparation method	Swift	TruSeq	QIAseq	Swift	TruSeq	QIAseq
DNA input (ng)	200			50		
DNA fragmentation before bisulfite treatment	Yes	No		Yes	No	
Bisulfite conversion kit	EZ DNA Methylation-Gold Kit		EpiTect Fast Bisulfite Kit	EZ DNA Methylation-Gold Kit	EpiTect Fast Bisulfite Kit	
Sequencing Platform	HiSeq X			HiSeq X		
Spike-in (%)	PhiX (5%)			12%~89.6% WGS sequences		
PCR enzyme	Enzyme R3	FailSafe	VeraSeq Ultra	Enzyme R3	FailSafe	VeraSeq Ultra
PCR cycles	7	10	9	9	12	6
Number of effective lanes per sample	1	1	1	1.17	1.13	1.15
Average data output per sample^1,2^	150.88 Gb, 499.61 Mrp	142.14 Gb, 470.65 Mrp	147.56 Gb, 488.61 Mrp	157.49 Gb, 524.95 Mrp	151.95 Gb, 506.49 Mrp	102.14 Gb, 340.45 Mrp

^1^Gb: giga base. Mrp: million read pair.

^2^In these experiments, one lane of HiSeq X flow cell generates 145.68 ± 9.96 (mean ± standard deviation (SD)) Gb from provider A, and 119.29 ± 24.50 Gb from provider B.

One lane of HiSeq X generates 100–125 Gb of data on average, which in turn is expected to yield ~33x genome coverage assuming no data loss during pre-processing. As data loss is expected due to various quality filters, the two sequencing providers determined the number of effective lanes to use by aiming for an estimated effective genome coverage of ~30x based on their respective experience and usual spike-in as well as pooling strategies.

One particular challenge in performing WGBS is the unbalanced base composition of the library. As early versions of the Illumina software were not designed to handle unbalanced libraries, a substantial spike-in of DNA of an unbiased composition, typically PhiX spike-in at ~25%, was necessary to maximise the cluster passing filter during sequencing and generate data of reasonable quality. With recent iteration of the HiSeq X software (Real-Time Analysis (RTA) 2.7.7 and HiSeq Control Software (HCS) 3.4.0.38), which includes a revised Q-table to facilitate sequencing of unbalanced libraries, a 5% spike-in of PhiX is now sufficient to generate high-quality sequencing data, which was the protocol adopted by Provider A. For Provider B, in line with their usual practise, their preferred strategy was to use Whole Genome Sequencing (WGS) spike-in instead (Table 1).

#### Comparison of quality metrices

**Figure 1:**
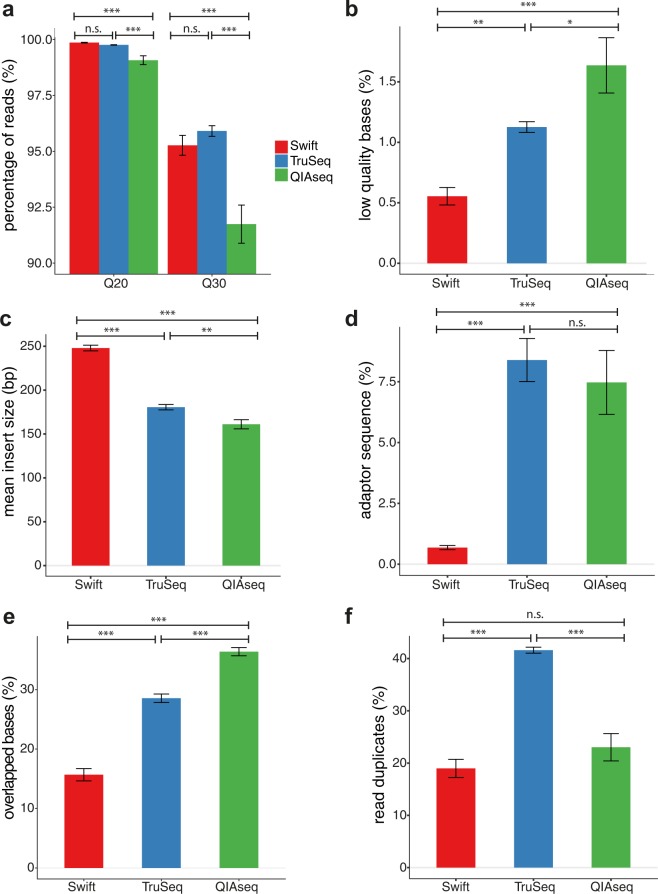
Comparison of quality metric across library preparation methods. (**a**) Raw reads with sequencing quality > Q20 or >Q30. (**b**) Bases trimmed due to low quality (<Q20). (**c**) Library insert size (bp). (**d**) Bases containing adaptor sequences. (**e**) Overlapping bases between Read 1 and Read 2. (**f**) Read duplicates (read-pairs). Read duplicates were defined as two read-pairs with the same start and end positions. All libraries were sequenced on the HiSeq X platform. n.s.: not statistically significant, P > 0.05, *P < 0.05, **P < 0.01, ***P < 0.001. Bars show average values, with error bars representing standard error of mean.

Now, we first examined the fraction of reads that have average sequence quality score of at least Q20 (Phred quality score of 20, equivalent to the probability of an incorrect base call 1 in 100) or Q30 across the three library preparation methods. Q20 fractions were similar between Swift and TruSeq, but significantly lower for QIAseq (Fig. 1a; Swift: 99.9%, TruSeq: 99.8%, QIAseq: 99.1%; P[ANOVA] = 2.21E-5; Swift vs. TruSeq: P = 7.34E-1, Swift vs. QIAseq: P = 5.71E-5, TruSeq vs. QIAseq: P = 1.09E-4). Results were similar at Q30 (Fig. 1a, Swift: 95.3%, TruSeq: 95.9%, QIAseq: 91.7%; P[ANOVA] = 1.02E-5; Swift vs. TruSeq: P = 6.38E-1, Swift vs. QIAseq: P = 3.15E-4, TruSeq vs. QIAseq: P = 1.00E-5). This was also true when we analyzed Read 1 (R1) and Read 2 (R2) separately (Supplementary Fig. 1a). As expected, the proportion of bases trimmed for low quality (Q < 20) was lowest for Swift and highest for QIAseq method in the overall libraries (Fig. 1b; Swift: 0.6%, TruSeq: 1.1%, QIAseq: 1.6%; P[ANOVA] = 2.33E-5; Swift vs. TruSeq: P = 6.89E-3, Swift vs. QIAseq: P = 1.39E-5, TruSeq vs. QIAseq: P = 1.62E-2), as well as in R1 and R2 raw reads respectively (Supplementary Fig. 1b). Consistent with previous studies, a higher proportion of bases were trimmed for low quality in R2 relative to R1 across all kits except for QIAseq, which is likely attributable to the larger variation observed (Supplementary Fig. 1b; Swift: P = 8.86E-5; TruSeq: P = 2.35E-9; QIAseq: P = 6.93E-1)^11^.

We next compared the insert size between the three library preparation methods. On average, insert sizes were significantly higher for libraries generated by the Swift method than those from TruSeq, and lowest for libraries by QIAseq (Fig. 1c; Swift: 248 bp, TruSeq: 181 bp, QIAseq: 161 bp; P[ANOVA] = 8.33E-14; Swift vs. TruSeq: P = 6.12E-12, Swift vs. QIAseq: P = 1.96E-13, TruSeq vs. QIAseq: P = 3.14E-3). In keeping with this, both TruSeq and QIAseq library preparation methods generated reads with high proportion of adaptor contamination, indicative of adaptor read-through (Fig. 1d; adaptor sequence trimming rates: Swift: 0.7%, TruSeq: 8.4%, QIAseq: 7.5%; P[ANOVA] = 8.02E-6; Swift vs. TruSeq: P = 9.20E-6, Swift vs. QIAseq: P = 1.82E-4, TruSeq vs. QIAseq: P = 7.59E-1). This distribution in terms of adaptor contamination was also observed in both R1 and R2 across all three methods, with no significant differences between R1 and R2 (Supplementary Fig. 1c). Since read pairs with insert size less than the length of a single read can read into the adaptor (adaptor read-through), we indeed observed here that adaptor trimming rate was significantly inversely correlated with insert size (Pearson’s r = −0.87, P = 2.11E-9). Also, as expected, shorter insert size was associated with higher proportion of overlapping bases between R1 and R2 (r = −0.98, P = 2.07E-20), with the proportion of overlapping bases between paired reads lowest for Swift, and highest for QIAseq method (Fig. 1e, Swift: 15.7%, TruSeq: 28.5%, QIAseq: 36.4%; P[ANOVA] = 4.39E-15; Swift vs. TruSeq: P = 6.91E-11, Swift vs. QIAseq: P = 4.74E-14, TruSeq vs. QIAseq: P = 8.81E-7).

Another measure of library quality is read duplication rate, with higher duplication rates indicative of lower library diversity^18^. Our results showed that the TruSeq library preparation method generated a higher fraction of duplicates relative to both Swift and QIAseq (Fig. 1f, Swift: 19.0%, TruSeq: 41.6%, QIAseq: 23.0%; P[ANOVA] = 2.00E-10; Swift vs. TruSeq: P = 7.31E-10, Swift vs. QIAseq: P = 2.41E-1, TruSeq vs. QIAseq: P = 3.41E-8).

#### Comparison of alignment rate, coverage depth and bias

**Figure 2:**
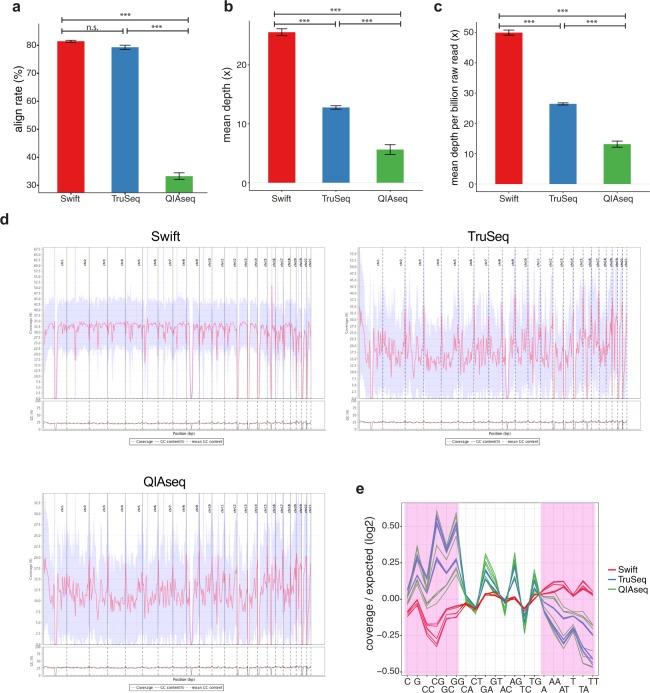
Comparison of alignment rate, depth and nucleotide amplification bias across library preparation methods. (**a**) Alignment rate. (**b**) Effective sequencing depth. Depth was calculated based on mapped reads after read duplicates removal and with overlapping bases counted only once. (**c**) Average depth per billion raw read pairs, calculated as mean depth divided by corresponding library size. (**d**) Depth distribution (coverage) across the genome. One representative sample (Sample 1) was plotted for each of the three methods, with coverage from only autosomal regions displayed. The blue shaded area indicates the standard deviation of depth within each bin (n = 421). The genome was divided into a total of 421 bins according to default settings by *qualimap*. (**e**) Nucleotide amplification biases, expressed as the logarithm 2 transformed ratio of observed to expected coverage for different nucleotide and dinucleotide combinations. G/C-rich category (defined as C, G, CC, CG, GC and GG) and A/T-rich category (defined as A, AA, AT, T, TA and TT) are highlighted in pink. All libraries were sequenced on the HiSeq X platform. Each line represents one sample (library). n.s.: not statistically significant, P > 0.05, *P < 0.05, **P < 0.01, ***P < 0.001. Bars show average values, with error bars representing standard error of mean.

We next compared mapping efficiency across the three library preparation methods. There was no difference between Swift and TruSeq in terms of mapping efficiency (Fig. 2a; Swift: 81.5%, TruSeq: 79.3%; Swift vs. TruSeq: P = 1.71E-1). QIAseq suffered the lowest mapping efficiency, with only one-third of its reads mapping successfully (Fig. 2a; Swift vs. QIAseq: P = 3.28E-14, TruSeq vs. QIAseq: P = 3.28E-14). A closer inspection of the unmapped reads from data generated by the QIAseq method revealed that on average, 13% (SD: 1.7%) were broken pairs and 51% (SD: 3.0%) were reads containing too many mismatches/deletion/insertion and/or other unknown sequences, both of which were not permitted during alignment (see Methods for details on mapping criteria).

We now turn to investigate the impact of the above observations on effective read depth. We applied the quality control and trimming parameters in line with previous reports, followed by alignment (Supplementary Fig. 2). We obtained an average effective read depth (mean depth) of 26x (SD: 2x) for Swift, followed by 13x (SD: 1x) for TruSeq, and finally, 6x (SD: 2x) for QIAseq (Fig. 2b; P[ANOVA] = 4.43E-18; Swift vs. TruSeq: P = 4.65E-14, Swift vs. QIAseq: P = 3.28E-14, TruSeq vs. QIAseq: P = 3.54E-9). Despite the similar alignment rates, libraries generated by the TruSeq method delivered read depths that were approximately 50% lower than that for the Swift method. This difference is accounted for by the higher proportion of low quality, duplicate and/or overlapping reads in the TruSeq compared to the Swift libraries (Fig. 1). To account for any potential bias due to differences in raw sequencing output, we normalize the effective depths by raw read counts (i.e. read counts before any of the above quality filter was applied). As expected, the Swift method achieved the highest normalized average read depth (mean depth per billion raw read; calculated as average effective read depth/library size) (50x per billion read pairs, SD: 2x), followed by TruSeq and QIAseq (26x (SD: 1x) and 13x (SD: 3x), respectively) (Fig. 2c; P[ANOVA] < 2E-16; Swift vs. TruSeq: P = 3.28E-14, Swift vs. QIAseq: P = 3.28E-14, TruSeq vs. QIAseq: P = 1.35E-12).

We then examined coverage across the reference genome by chromosome to determine if there was any bias in the distribution of reads across and within the chromosomes (Fig. 2d). The Swift method presented the most uniform coverage across the genome (P = 1.00E+0, Chi-Square goodness of fit test), followed by QIAseq (P = 3.70E-1), with the distribution for TruSeq significantly non-uniform (P = 3.05E-49). We also noted ‘0’ coverage at gap regions (‘N’s) across all three library preparation methods. In addition, we observed the TruSeq method resulted in clear biases in the form of GC-rich regions enrichment/AT-rich regions depletion across all nucleotide categories, while this bias was less obvious with the other two library preparation methods (Fig. 2e, Supplementary Table 1). In addition, we also observed that the Swift method was the only approach that did not display significant differences in nucleotide amplification bias between our two providers (Supplementary Table 1).

**Figure 3:**
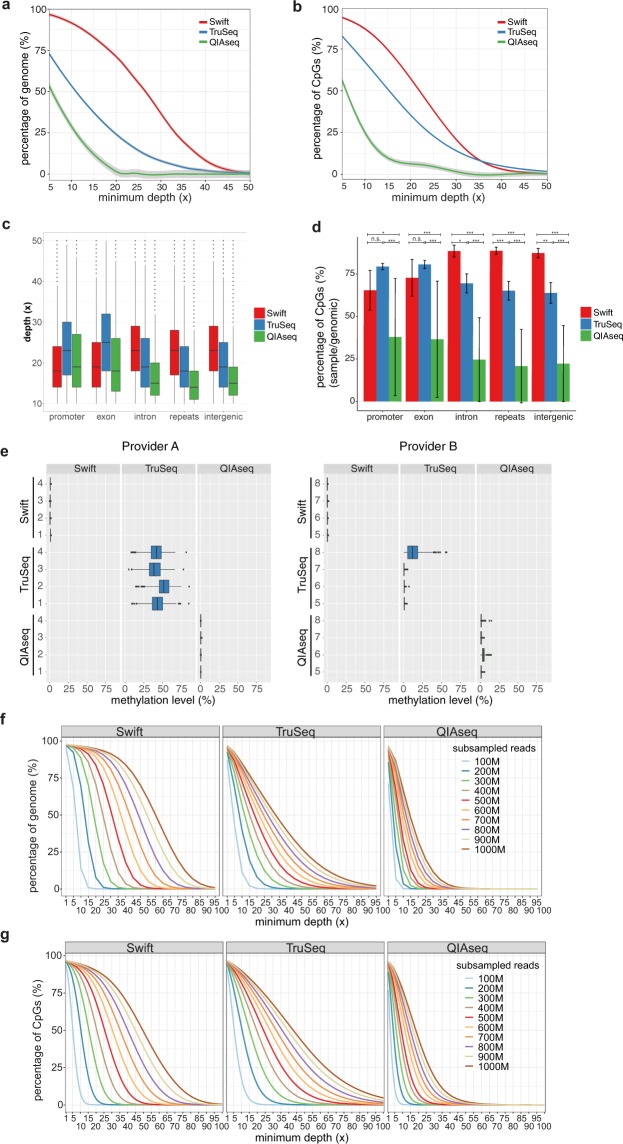
Comparison of genome and CpG coverage at different minimum depths and genomic regions across library preparation methods. (**a**) Genome coverage at different minimum depths (5–50x). (**b**) CpG site coverage at different minimum depths (5–50x). For (**a**,**b**), Loess smoothing was applied, with the 95% confidence intervals indicated by gray shaded areas. (**c**) Distribution of depth (coverage) for CpG sites across different genomic features. Only CpGs with minimum depth between 10x and 50x (~99% of all CpG sites) were displayed in the boxplots. (**d**) Average percentages of CpGs covered at different genomic features. The percentages were calculated by dividing the number of CpG sites covered with a minimum depth of 10x for each genomic feature by the total number of CpG sites in the genome for the corresponding genomic feature. (**e**) Boxplots of methylation level for CpGs in the mitochondria across all libraries by Provider A (left) and B (right). Only CpGs with a minimum depth of 10x were included. (**f**) Percentage of genome covered at each minimum depth across different number of raw read pairs. (**g**) Percentage of CpG sites in the genome covered at each minimum depth across different number of raw read pairs. For (**f**,**g**), results obtained from downsampling analyses are shown here for minimum depths ranging from 1–100x for library sizes consisting of 100–1000 M raw read pairs. All available samples were pooled for the downsampling analyses. All libraries were sequenced on the HiSeq X platform. n.s.: not statistically significant, P > 0.05, *P < 0.05, **P < 0.01, ***P < 0.001. Error bars represent standard error of mean.

We next compared the percentage of genome that is covered at each minimum depth cutoff across the three methods. In line with the mapping rates observed, we found that the Swift method covered a significantly higher percentage of the genome relative to the other two methods up to 40x, after which the difference became insignificant between Swift and TruSeq, with QIAseq remaining the worst performing method across the entire range of minimum depths (Fig. 3a, Supplementary Table 2).

A similar trend was observed when we focused on only CpG sites coverage across the genome, with the Swift method again outperforming the other two library preparation methods at minimum depths below 35x. However, at cutoffs above 35x, TruSeq covered the highest percentage of CpG sites in the human genome, although the difference was marginal, not always statistically significant relative to the Swift method and was observed at less than 8% of CpGs (Fig. 3b, Supplementary Table 3). When stratified by genomic feature, the Swift method showed that CpG sites from intron, repeat and intergenic regions were covered at higher depth relative to those from promoter and exon regions, whilst the opposite was true for both TruSeq and QIAseq methods (Fig. 3c, P < 0.001). This resulted in the Swift method delivering successful quantification of methylation at 10x for a higher proportion of CpG sites IN intron, repeat and intergenic regions, compared to alternate approaches (Fig. 3d).

When examining the distribution of methylation level across the chromosomes, consistent with previous reports^19,20^, we found very low methylation signal in mitochondrial DNA (mtDNA) with Swift and QIAseq (Fig. 3e; Supplementary Table 4). For TruSeq, methylation signals were high (44.1% ± 5.3%) for libraries prepared by provider A, but very low for those by provider B (3.8% ± 6.3%). This is likely due to differences in DNA input, whereby Provider A did not perform any form of DNA fragmentation prior to bisulfite conversion, while Provider B sonicated the input DNA.

To determine the raw library size (i.e. number of read pairs before applying quality filters) needed to achieve a prespecified percentage of genome or CpG coverage at a certain minimum read depth, we performed downsampling analysis on our current dataset, utilizing all available sequencing data across our samples. The percentage of genome and CpGs covered at different minimum depths across a range of raw library sizes were interrogated for the three library preparation methods (Fig. 3f,g). Our results suggest that at least 500 million raw library reads (pair-end reads) would be necessary to achieve 50% genome coverage at minimum depth of 30x with the Swift method, whereas 900 million and more than one billion raw read pairs will be needed for TruSeq and QIAseq respectively (Fig. 3f). Similarly, in terms of CpG coverage, at least 600 million raw read pairs would be required to achieve 50% CpG coverage at minimum depth of 30x with the Swift method, whereas 700 million and more than one billion raw read pairs were necessary for TruSeq and QIAseq respectively (Fig. 3g).

### Comparison between NovaSeq and HiSeq X sequencing platforms

**Figure 4:**
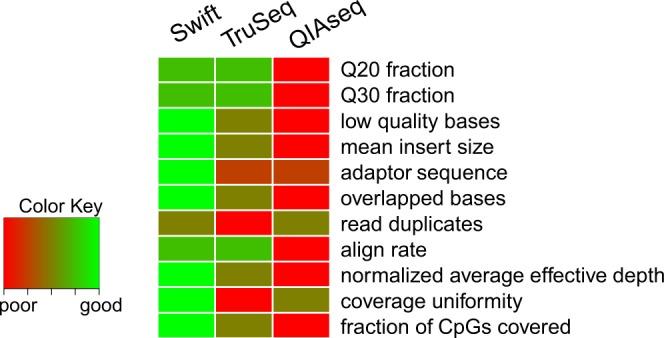
Overall performance comparison between library preparation methods. Heatmap displaying rankings of the three library preparation methods by main performance metric. The libraries are ranked from one to three, with one (red) for the worst performing method and three (green) for the best performing method. Where there is no statistically significant difference between any pairwise comparison for a particular metric, the two methods will be given the same rank equivalent to the average ranks e.g. for Q20 fraction, QIAseq was the worst performing method (hence given a rank score of 1), while both Swift and TruSeq had the same rank score of 2.5 = (2 + 3)/2 since there was no statistically significant difference between the two.

Having systematically evaluated the performance of the three library preparation methods, we next compared performance between the two leading Illumina high-throughput sequencing platforms: NovaSeq 6000 launched early 2017 and the more commonly available HiSeq X platforms (Table 2). Relative to the HiSeq X, the NovaSeq provides more reads per run and a higher maximum output by virture of its higher cluster density. The run time is also shorter on the NovaSeq, primarily achieved via the migration to the two-color chemistry from the four-dye system. For this section, all libraries were generated based on the best-performing protocol established above (bisulfite conversion: EZ DNA Methylation-Gold Kit; library preparation: Swift (Fig. 4)), with the same library split into two and sequenced independently on NovaSeq 6000 and HiSeq X.

**Table 2:** Experimental setup for comparison of sequencing platforms.

Provider	A		B	
Samples	Samples 1–4		Samples 5–8	
Sample Type	Whole Blood		Isolated cell subtypes	
Library Preparation Method	Swift			
DNA input (ng)	200		50	
DNA fragmentation before bisulfite treatment	Yes			
Bisulfite conversion kit	EZ DNA Methylation-Gold Kit			
Sequencing Platform	HiSeq X	NovaSeq	HiSeq X	NovaSeq
Spike-in (%)	PhiX (5%)		WGS sequences (12~89.6%)	WGS sequences (86~96%)
PCR enzyme	Enzyme R3			
PCR cycles	7		9	
Effective number of lanes per sample	1	0.25	1.17	0.56
Average data output per sample^1,2^	150.88 Gb, 499.61 Mrp	226.95 Gb, 751.50 Mrp	157.49 Gb, 524.95 Mrp	250.91 Gb, 836.38 Mrp

^1^Gb: giga base. Mrp: million read pair.

^2^In these experiments, one lane of HiSeq X flow cell generates 150.88 ± 0.82 (mean ± SD) Gb from provider A, and 134.75 ± 17.31 Gb from provider B. One lane of NovaSeq S4 flow cell generates 907.81 ± 103.92 Gb from provider A, and one lane of S2 flow cell generates 448.06 ± 60.07 Gb from provider B.

Library preparation and sequencing procedures were carried out by two independent sequencing providers according to their respective best practice. One lane of NovaSeq S2 and S4 flow cell is expected to produce on average 500–625 Gb and 600–750 Gb of data respectively. In line with the data generation predicted, the anticipated effective genome coverage per lane was ~30x for the HiSeq X sequencing, and ~160x and ~200x for the NovaSeq platform for S2 and S4 flow cell respectively (Table 2).

#### Comparison of quality metrices

**Figure 5:**
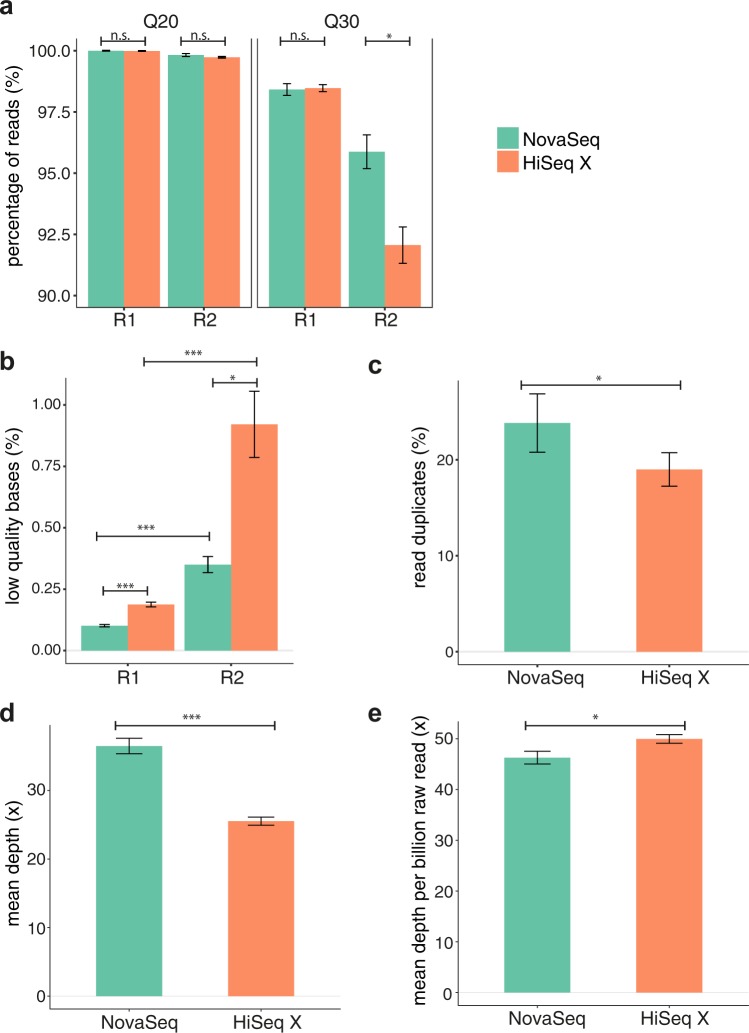
Comparison of quality metric for libraries sequenced on the NovaSeq and HiSeq X sequencing platforms. (**a**) Raw reads with sequencing quality > Q20 or > Q30. Values from Read 1 and Read 2 were analyzed separately. (**b**) Bases trimmed due to low quality (<Q20). (**c**) Read duplicates (read-pairs). Read duplicates were defined as two read-pairs with the same start and end positions. (**d**) Effective sequencing depth, calculated based on mapped reads after read duplicates removal, with overlapping bases counted only once. (**e**) Average depth per billion raw read pairs. All libraries were generated by the Swift library preparation kit. The same libraries were split into two and sequenced independently on NovaSeq 6000 and HiSeq X. n.s.: not statistically significant, P > 0.05, *P < 0.05, **P < 0.01, ***P < 0.001. Bars show average values, with error bars representing standard error of mean.

For R1 raw reads, there were no significant difference between NovaSeq and HiSeq X in terms of both Q20 and Q30 fractions (Fig. 5a; Q20 NovaSeq: 100.0%, HiSeq X: 99.9%; P > 0.05; Q30 NovaSeq: 98.4%, HiSeq X: 98.5%, P > 0.05). However, for R2, although there was no significant difference for Q20, we observed a significantly higher Q30 fraction for NovaSeq (Fig. 5a; Q20 NovaSeq: 98.8%, HiSeq X: 98.7%, P = 3.49E-1; Q30 NovaSeq: 95.9%, HiSeq X: 92.1%, P = 3.10E-2). Similarly, the fraction of bases trimmed for low quality was also smaller for NovaSeq relative to HiSeq X for both R1 and R2 (Fig. 5b**;** R1 NovaSeq: 0.1%, HiSeq X: 0.2%, P = 2.94E-5; R2 NovaSeq: 0.4%, HiSeq X: 0.9%, P = 1.07E-2). As observed in the previous section as well as earlier studies^11,21,22^, a higher proportion of low quality bases were trimmed for R2 compared to R1 on both platforms (Fig. 5b; NovaSeq: P = 1.31E-4; HiSeq X: P = 6.28E-4). Higher read duplication rate was also observed on the NovaSeq compared to the HiSeq X (Fig. 5c; NovaSeq: 23.8%, HiSeq X: 19.0%; P = 1.60E-2).

#### Comparison of coverage depth and bias

As expected, sequencing output was higher for the NovaSeq compared to the HiSeq X platform (Table 2; NovaSeq 239 Gb per sample (SD: 31 Gb) vs. HiSeq X 154 Gb per sample (SD: 14 Gb); P = 3.34E-5). Mean effective read depth for NovaSeq and HiSeq X sequencing were 37x  ± 3x (SD) and 26x  ± 2x respectively (Fig. 5d; P = 4.21E-5). After normalization for sequencing output, NovaSeq now had a lower effective read depth (46x per billion read pairs ± 4x), compared to HiSeq X (50x per billion read pairs ± 2x) (Fig. 5e, P = 1.22E-3). This difference was primarily driven by the higher duplication rate observed with the NovaSeq platform (Fig. 5c).

**Figure 6:**
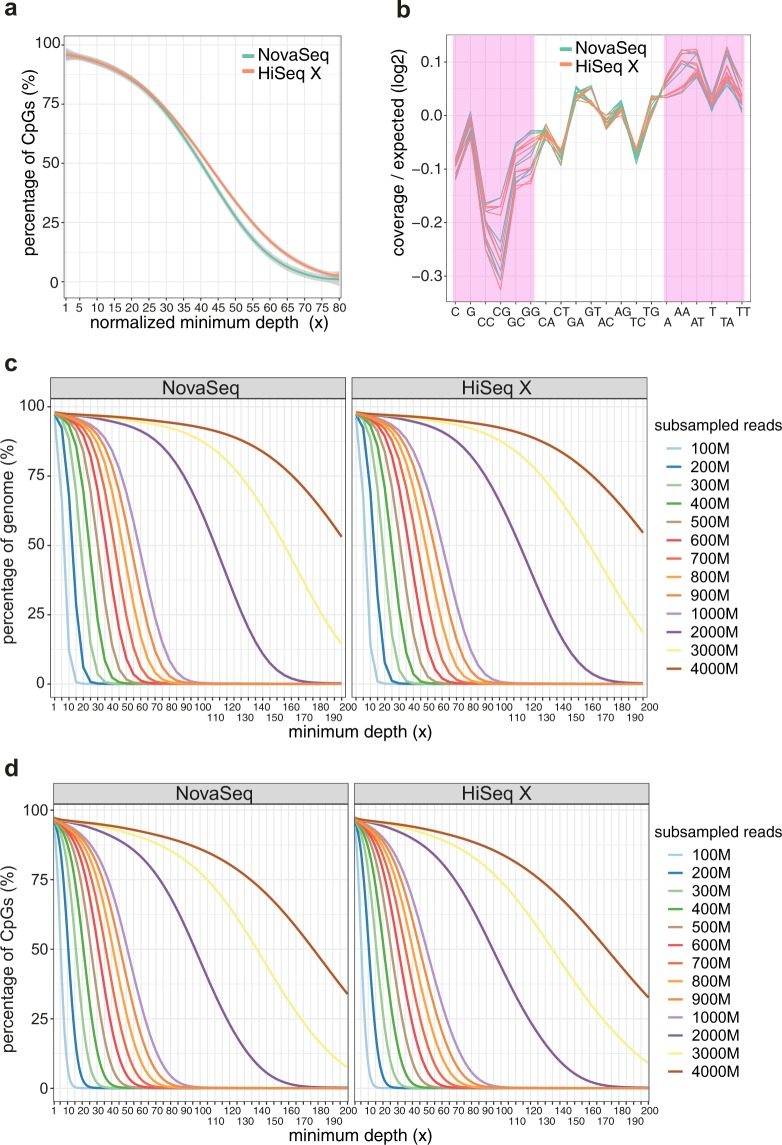
Comparison of genome and CpG coverage at different minimum depths and nucleotide amplification bias between NovaSeq and HiSeq X sequencing platforms. (**a**) CpG site coverage at different minimum depths (1–80x). Loess smoothing was applied, with the 95% confidence intervals indicated by gray shaded areas. (**b**) Nucleotide amplification biases, expressed as the logarithm 2 transformed ratio of observed to expected coverage for different nucleotide and dinucleotide combinations. G/C-rich category (defined as C, G, CC, CG, GC and GG) and A/T-rich category (defined as A, AA, AT, T, TA and TT) are highlighted in pink. Each line represents one sample (library). (**c**) Genome coverage at each minimum depth across different number of raw read pairs. (**d**) CpG site coverage at each minimum depth across different number of raw read pairs. For **c** and **d**, results obtained from downsampling analyses are shown here for minimum depths ranging from 1–200x for library sizes consisting of 100–4000 M raw read pairs. All available samples were pooled for the downsampling analyses. n.s.: not statistically significant, P > 0.05, *P < 0.05, **P < 0.01, ***P < 0.001. Bars show average values, with error bars representing standard error of mean.

We next compared the percentage of total CpG sites covered at different minimum depths (1–80x) between NovaSeq and HiSeq X platforms after normalizing for the sequencing output and scaled up to one billion read pairs. In general, there was no statistical difference in the coverage between NovaSeq and HiSeq X at normalized minimum depth less than 45x (Fig. 6a; Supplementary Table 7). In terms of nucleotide amplification bias, no significant difference was observed between the two platforms (Fig. 6b, Supplementary Table 8; P > 0.05).

Lastly, we performed downsampling analysis on samples analyzed on the two sequencing platforms. For the two platforms, a similar number of paired-end reads is needed to achieve a comparable mean sequencing depth, as well as similar CpG coverage at any given minimum depth (Fig. 6c,d; Supplementary Tables 9–10). Both normalization and downsampling confirmed that genome coverage was similar between the NovaSeq and HiSeq X platforms at comparable sequencing output.

### Comparison between WGBS and array data

Next, we evaluated the performance of WGBS for quantification of DNA methylation, in comparison to established methods based on methylation arrays. For WGBS, we focused on results generated using Swift library preparation method and HiSeq X platform as the optimal combination based on our earlier experiments. Samples 1–4 were assessed on the Illumina Infinium HumanMethylation450 assay (450K)^23^, while Samples 5–8 were assessed on the MethylationEPIC BeadChip (EPIC) assay^24^. DNA methylation level was quantified based on previously established workflow as outlined in Supplementary Fig. 2 for WGBS and by the CPACOR pipeline for methylation arrays^25^.

**Figure 7:**
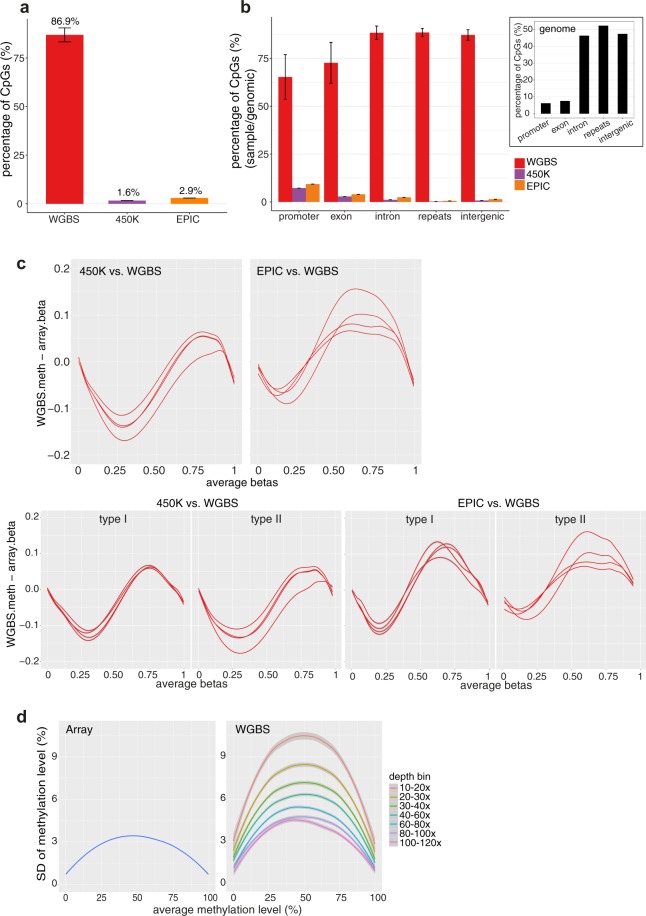
Comparison of performance between WGBS and methylation arrays on the HiSeq X sequencing platform. (**a**) CpG site coverage by WGBS and methylation arrays (450 K and EPIC). (**b**) CpG site coverage at different genomic features. The percentages were calculated by dividing the number of CpG sites covered with a minimum depth of 10x for each genomic feature by the total number of CpG sites in the genome for the corresponding genomic feature. Inset: Distribution of CpG sites in the genome by genomic features. Bars show average values, with error bars representing standard error of mean. (**c**) Bland-Altman plots comparing methylation levels reported by WGBS and the methylation arrays. Upper (all probe types): WGBS versus 450 K (left), WGBS versus EPIC (right). Lower (stratified by Type I and Type II probes): WGBS versus 450 K (left), WGBS versus EPIC (right). Each line represents one sample (library). (**d**) Comparison of standard deviation (SD) of methylation levels of replicate samples between WGBS and methylation arrays. CpG sites were binned according to their average coverage, inclusive of the lower limit and exclusive of the upper limit. For WGBS data, only CpG sites with a minimal depth of 10x were used across all analyses shown here. For **c** and **d**, only CpG sites found in both WGBS and array data are considered in the analyses. All WGBS data included in this analysis are generated by the Swift library preparation kit on the HiSeq X platform. For comparison of performance between WGBS and methylation arrays across all three library preparation methods on the HiSeq X platform, including TruSeq and QIAseq, see Supplementary Fig. 3a–d**;** Supplementary Table 11. For comparison of performance between WGBS and methylation arrays with the Swift method on both HiSeq X and NovaSeq platforms, see Supplementary Fig. 4a–d. n.s.: not statistically significant, P > 0.05, * P < 0.05, ** P < 0.01, ***P  < 0.001.

We found that while the methylation arrays were able to assay only <3% of all CpG sites in the human genome, WGBS quantifies methylation at the great majority of CpG sites (87% at 10x coverage; Fig. 7a). WGBS also yielded considerably higher CpGs coverage within each genomic feature relative to both arrays (Fig. 7b).

We next evaluated the concordance between methylation levels assayed by WGBS and array. The analysis was restricted to only CpG sites available on both platforms, and CpG sites with a minimal depth of 10x coverage with WGBS. For these CpG sites, an average read depth of 24x (SD: 1.4x) was achieved. As expected, we observed global correlation levels to be high between array and WGBS, with correlation coefficients ranging from 0.95 to 0.97 (Supplementary Fig. 3). In addition, as observed from the Bland-Altman plots^26,27^, for CpG sites with beta values between 0 and 0.5, on average, methylation levels from WGBS were 6.78% (SD:1.06%; P = 1.03E-3) and 3.19% (SD:1.00%; P = 7.66E-3) lower than beta values from the 450 K and EPIC methylation array respectively (Fig. 7c). In contrast, for beta values between 0.5 and 1, WGBS rendered methylation levels that were higher than the arrays by 2.76% (SD:1.43%; P = 3.09E-2) and 7.68% (SD:2.65%; P = 1.02E-2) for 450 K and EPIC respectively (Fig. 7c). The bias between WGBS and array was similar for Type I and Type II probes^23,24^ (Fig. 7c; P = 5.73E-1 and 3.73E-1 for 450 K and EPIC respectively) and across a range of read depths (Supplementary Fig. 4; P = 0.998 and P = 1 for 450 K and EPIC respectively).

To further compare the reproducibility of WGBS and methylation array data, we quantified variability for technical replicates (n = 2) of each biological sample. Array showed higher precision than WGBS across the entire assay range, with precision improving at higher sequencing depth. As expected, variation was lower for measurements at CpG sites covered at higher depth for WGBS (Fig. 7d; Supplementary Table 11). At the current recommended coverage of 30x^15^, the standard deviation (SD) observed for WGBS was two to three fold higher than that on the methylation array. This suggests that to achieve a level of precision that is broadly similar to that observed in methylation array, a coverage of at least 100x will be necessary.

To illustrate the impact of the relationship between depth and precision, we calculated sample sizes needed for population-based case-control studies using WGBS or methylation array across a range of differences in methylation levels, taking into account both technical and biological variability (Supplementary Table 12). Compared to array, the sample sizes needed to identify comparable changes in methylation are similar using 100x WGBS, but three times greater using 30x WGBS sequencing.

## Discussion

In this study, we set out to systematically evaluate three contemporary WGBS library preparation methods across two state-of-the-art sequencing platforms. We also assessed the performance and precision of the WGBS method relative to the methylation arrays, in an effort to provide data-driven recommendations for future WGBS studies, in particularly with respect to minimum coverage.

Traditional WGBS library preparation workflow involves DNA fragmentation, sequencing adaptor ligation prior to bisulfite treatment and Polymerase Chain Reaction (PCR) amplification^7,28^. As large amounts of DNA are typically lost during fragmentation and bisulfite conversion, this renders WGBS less suitable for many human studies whereby samples are often limited in quantity. Here, we focus on three commercially available post-bisulfite library preparation methods which requires lower DNA input^16,17,29^.

Our results showed the Swift method outperforms the other two library preparation methods across various metrics, including percentage of low quality bases, read duplicate rates and coverage uniformity, which is potentially attributable to differences in underlying chemistry such as the polymerase used. The Swift method also achieved the highest effective read depth, with significantly higher percentages of both genome and CpG sites interrogated across all depths. One reason for this may be the larger insert size for libraries generated by the Swift method. Longer inserts benefit from less adaptor contamination and overlapping reads, both of which increases coverage efficiency^30^. This could potentially be accounted for by the lower volumetric ratio of magnetic beads to DNA used by the Swift method (Swift: 0.85; TruSeq: 1.0; QIAseq: 1.6), which preferentially enrich larger fragments in the final cleanup step during library preparation^9,31^.

In addition, the TruSeq protocol contains the highest number of PCR amplification cycles (10–12 cycles), in contrast to only 7–9 and 6–9 cycles for Swift and QIAseq respectively. This might help explain the higher duplication rate with the TruSeq method, and hence reduce downstream library diversity and genome coverage, although the impact is minimal beyond ~10 cycles^32,33^. The high number of PCR cycles in the TruSeq method is also in line with the observation that its libraries showed the highest degree of nucleotide amplification/GC bias. GC bias in sequencing can occur due to factors such as priming, size selection, and probability of sequencing errors, with PCR previously identified as the main contributor^34–40^. The observed GC bias is clearly a disadvantage as it impacts upon even read coverage, which was observed with the TruSeq method.

Indeed, the Swift method was able to cover significantly higher percentages of the genome as well as CpG sites relative to the other two methods. CpG sites from intron, repeats and intergenic regions, typically with lower GC content, were covered at a higher depth relative to those from the promoter and exon regions by the Swift method, while the opposite trend was true for TruSeq and QIAseq methods. This suggests that the Swift method could cover more potentially functionally relevant CpG sites in intron, repeats and intergenic regions than the other two methods^41–44^.

We also systematically evaluated the performance of the two leading Illumina high-throughput sequencing platforms, namely HiSeq X and NovaSeq. We observed a higher read duplication rate on the NovaSeq platform. This might reflect the shorter distance between the nanowells on the NovaSeq flow cell, which has been proposed to increase the probability of forming another cluster in a neighboring nanowell from the same original DNA molecule during cluster generation, which is commonly referred to as Exclusion Amplification (ExAmp) duplicates^45,46^. Higher cluster densities on the flow cell has also been suggested to suppress GC-rich reads^39^. However, we did not observe any differences in GC bias between the two platforms.

Data quality was comparable between HiSeq X and NovaSeq, with a marginally higher Q30 fraction on R2 and lower proportion of low quality bases on the NovaSeq. This may seem counter-intuitive, given that the two-color chemistry utilized on the NovaSeq has been previously suggested to overcall high confidence G bases due to its inability to distinguish between no signal and a G base^47^. However, it is worth noting that this problem with false G bases, in particular with poly-G tails, is much less prominent when overall data quality is high, as was the case here in our study.

This marginal improvement in data quality and reduction in workflow time on the NovaSeq appears to come at a cost. Even on the most cost efficient NovaSeq configuration (i.e. NovaSeq 6000 with S4 flow cell), the cost per Gb of data generated remains higher than that on the HiSeq X. Nonetheless, as the NovaSeq has only been recently launched, it is expected that its cost of sequencing will decrease over time as the associated experimental protocol gets optimized.

One key advantage in the utilization of WGBS is its ability to provide unbiased genomewide coverage. Indeed, WGBS method, as expected, quantified methylation at substantially more CpG sites than methylation arrays (24.6 millions versus 0.8 million out of 28.7 million). The improvement in coverage was most notably for introns, repeats and intergenic regions which are not well represented in the available methylation arrays, and which have been suggested to be key locations for functionally important CpG sites^44^.

We also observed systematic biases in methylation levels between WGBS and methylation arrays, likely due to differences in its underlying chemistry and mathematical approach to methylation quantification. WGBS quantifies methylation level based on discrete read counts, while the array measures fluorescence intensity detected from its probes. While array measurement has been established to be accurate and reproducible, the performance of WGBS as an assay for quatification of DNA methylation has not been widely studied.

We showed that at the recommended sequencing depth of 30x, the technical variability for WGBS is two to three fold higher than that for the methylation array. This lower precision observed is likely to be due to insufficient reads/counts to generate reliable quantification^48^. The precision of WGBS improves with increased sequencing depth as observed from our downsampling analyses, and a minimum coverage of 100x is necessary to achieve a level of precision that is broadly similar to that observed in methylation array. Given the differential cost for sequencing at different depths, this has important implications for the design of future population-based WGBS studies.

## Methods

### Samples from whole blood

Samples were obtained from participants of The London Life Sciences Prospective Population Study (LOLIPOP). LOLIPOP is a prospective cohort study of ~28 K Indian Asian and European men and women, recruited from the lists of 58 General Practitioners in West London, United Kingdom between 2003 and 2008^49–52^. Aliquots of whole blood collected at enrolment were stored at −80 °C for extraction of genomic DNA. The LOLIPOP study is approved by the National Research Ethics Service (NRES Committee London Fulham 07/H0712/150) and all experiments were performed in accordance with relevant guidelines and regulations. All participants gave written informed consent.

### Samples from isolated white blood cells

Samples from isolated white blood cells were obtained from DNA of individuals recruited at random from the outpatient departments at Ealing and University College Hospitals London as previously described^4^. Only DNA extracted from CD4^+^ T cell and neutrophils were used in this study. All experiments were performed in accordance with relevant guidelines and regulations. Informed consent was obtained from all participants (NRES Committee London Fulham 07/H0712/150, NRES Committee London City Road and Hampstead 13/LO/0477 and NRES Committee London Harrow 09/H0715/65).

### Library preparation and sequencing strategy

Fifty or 200 ng of genomic DNA was used for library preparation according to the respective sequencing providers’ usual practice. DNA was bisulfite converted with the EZ DNA Methylation Gold Kit (Zymo Research) for use with the Swift and TruSeq library preparation methods, while the EpiTect Fast bisulfite conversion kit (QIAGEN) was to be used with the QIAseq method according to manufacturers’ preferences. The libraries were subsequently generated according to their respective protocols^16,17^.

Briefly, for Swift library construction, adaptase was applied to the bisulfite-converted DNA, with adaptors as well as a low-complexity tail simultaneously ligated to the 3′ end of the DNA fragments. Following a primer extension, the bottom strand DNA was synthesized and the 3′ end was ligated with adaptor sequences. For TruSeq, bisulfite-treated DNA was first primed by random hexamers which carried a tag sequence on its 5′ end. Subsequently, the bottom strand DNA was extended and the 3′ end was annealed with another terminal-tagging oligo, which induce the synthesis of the 3′ adaptors. As for the QIAseq library preparation, bisulfite-converted DNA was repaired, primed with a short primer which induced bottom strand DNA synthesis, followed with end-polishing of the DNA strand and adaptor ligation.

Subsequently, all the above single-stranded DNA fragments with adaptors ligated were purified and enriched by Polymerase Chain Reaction (PCR) according to respective kits’ instruction manuals. The resulting PCR products were cleaned up and quality was assessed by an Agilent 2100 Bioanalyzer. Before sequencing, the library was spiked in with other high-complexity sequences, e.g. PhiX or WGS sequences according to the providers’ usual protocols to compensate for the reduced sequence diversity in bisulfite converted libraries. Finally, amplified DNA fragments were quantified by qPCR or PicoGreen assay and sequenced on either HiSeq X or NovaSeq 6000 platform to generate 2 × 150 bp paired-end libraries. The HiSeq Control Software (HCS) versions used were HCS:3.5.0.7 and HD 3.4.0.38, along with Real Time Analysis software version 2.7.7.

### Data quality analysis and processing

Raw sequencing output BCL data were converted to FASTQ files by using bcl2fastq v2.20 software^53^. The sequence quality was checked by FastQC v0.11.5^54^. Adaptors and low quality sequences (Phred <20) were trimmed by Trim Galore v0.4.4_dev and cutadapt v1.15^55,56^. Different additional trimming were applied according to the library preparation method used. As random oligos (6N) and low complexity tail (8N) introduced during TruSeq and Swift library preparation respectively would lead to artifactual cytosine methylation calling and low quality sequencing bases, nucleotide trimming is recommended^57,58^. We tested parameters with different levels of stringency to optimize the trimming criteria in an effort to strike a balance between mapping rates and depth of coverage. For the QIAseq method, although no length information of the oligos used for random priming was provided from the library preparation protocol, we similary tested different parameters at varying strigency and arrived at the current parameters which balances amount of data loss (coverage depth) and mapping efficiency. The final trimming parameters were as follows: Swift:–clip_R2 18–three_prime_clip_R1 18; TruSeq:–clip_R1 8–clip_R2 8–three_prime_clip_R1 8–three_prime_clip_R2 8; QIAseq:–clip_R1 10–clip_R2 10. Trimmed reads were checked by FastQC again before mapping to bisulfite-converted hg19 genome by bismark^30^.

To generate the bisulfite-converted reference genome, the hg19 genome sequences were firstly converted to both a C-to-T and a G-to-A version (equivalent to a C-to-T conversion on the reverse strand). Similarly, the sequencing reads obtained were also converted in the same fashion before alignment by bismark. The mapping parameters were: bismark –bowtie2 -p 4 –bam–score_min L,0,−0.2. The mapped reads were deduplicated by deduplicate_bismark and sorted by samtools v1.3 for further analysis^59^. Read duplicates were defined as two read-pairs with the same start and end positions. The depth of coverage distribution per chromosome was generated by Qualimap v2.2.1^60^. Read depth of coverage and insert size were analyzed by Picard tools v2.18.16^61^. The effective depth was calculated after removing read duplicates and counting the overlapping bases only once. DNA methylated sites were identified, extracted and counted by bismark_methylation_extractor. Methylation level were computed by custom-made R scripts. Only CpG sites with depth of coverage >10x were considered for methylation analysis. Methylation array analyses were performed according to the workflow published by Lehne *et al*.^25^. All data analyses were conducted by custom-made bash and R scripts (R version > = 3.4.4).

### Genomic features definition

All genomic features were defined based on hg19 genomic annotation Reference Sequence (RefSeq) database acquired from UCSC table browser^62^. Promoters were defined as regions of +/−1kb around transcription start sites^63^. Exonic and intronic regions were according to transcripts definition, after removing the promoter regions as defined above. Repeats regions were downloaded from UCSC table browser, after filtering for exonic, intronic and promoter regions as defined above. Intergenic region were defined as regions of the genome not overlapping with gene body, promoter or repeats regions as defined above.

### Statistical tests for comparisons between groups

One-way Analysis of Variance (ANOVA), followed by post-hoc Tukey HSD test were carried out to compare various performance metrices of the three library preparation methods (Swift, TruSeq and QIAseq). Paired t-test was used to assess the difference between NovaSeq and HiSeq X platforms. For other comparisons against average performance or null hypothesis of expected performance (e.g. no GC bias), t-test or Wilcoxon/Mann-Whitney U test was applied as appropriate. The chi-square test was used to test for uniformity of coverage, with observed distribution corresponding to mean coverage in individual bins and expected distribution corresponding to overall mean coverage after removal of regions with no coverage in the gap regions. For the Bland-Altman plot, the difference between WGBS sample methylation level and array beta value were plotted against the average array beta values.

## Declarations

### Ethics approval and consent to participate

The LOLIPOP study is approved by the National Research Ethics Service (NRES Committee London Fulham 07/H0712/150) and all participants gave written informed consent. All participants whereby samples were obtained from isolated white blood cells also gave written informed consent for inclusion in the study (NRES Committee London Fulham 07/H0712/150, NRES Committee London City Road and Hampstead 13/LO/0477 and NRES Committee London Harrow 09/H0715/65).

## Supplementary information

Dataset 1

Supplementary Information

## Data Availability

The datasets generated and/or analysed during the current study are available in the NCBI Gene Expression Omnibus (GEO; https://www.ncbi.nlm.nih.gov/geo) under accession number GSE128734.

## References

[1] Robertson KD (2005). DNA methylation and human disease. Nature Reviews Genetics.

[2] Smith ZD, Meissner A (2013). DNA methylation: roles in mammalian development. Nature Reviews Genetics.

[3] Chambers John C, Loh Marie, Lehne Benjamin, Drong Alexander, Kriebel Jennifer, Motta Valeria, Wahl Simone, Elliott Hannah R, Rota Federica, Scott William R, Zhang Weihua, Tan Sian-Tsung, Campanella Gianluca, Chadeau-Hyam Marc, Yengo Loic, Richmond Rebecca C, Adamowicz-Brice Martyna, Afzal Uzma, Bozaoglu Kiymet, Mok Zuan Yu, Ng Hong Kiat, Pattou François, Prokisch Holger, Rozario Michelle Ann, Tarantini Letizia, Abbott James, Ala-Korpela Mika, Albetti Benedetta, Ammerpohl Ole, Bertazzi Pier Alberto, Blancher Christine, Caiazzo Robert, Danesh John, Gaunt Tom R, de Lusignan Simon, Gieger Christian, Illig Thomas, Jha Sujeet, Jones Simon, Jowett Jeremy, Kangas Antti J, Kasturiratne Anuradhani, Kato Norihiro, Kotea Navaratnam, Kowlessur Sudhir, Pitkäniemi Janne, Punjabi Prakash, Saleheen Danish, Schafmayer Clemens, Soininen Pasi, Tai E-Shyong, Thorand Barbara, Tuomilehto Jaakko, Wickremasinghe Ananda Rajitha, Kyrtopoulos Soterios A, Aitman Timothy J, Herder Christian, Hampe Jochen, Cauchi Stéphane, Relton Caroline L, Froguel Philippe, Soong Richie, Vineis Paolo, Jarvelin Marjo-Riitta, Scott James, Grallert Harald, Bollati Valentina, Elliott Paul, McCarthy Mark I, Kooner Jaspal S (2015). Epigenome-wide association of DNA methylation markers in peripheral blood from Indian Asians and Europeans with incident type 2 diabetes: a nested case-control study. The Lancet Diabetes & Endocrinology.

[4] Wahl S (2017). Epigenome-wide association study of body mass index, and the adverse outcomes of adiposity. Nature.

[5] Jorda M (2017). The epigenetic landscape of Alu repeats delineates the structural and functional genomic architecture of colon cancer cells. Genome Research.

[6] Stirzaker C, Taberlay PC, Statham AL, Clark SJ (2014). Mining cancer methylomes: prospects and challenges. Trends Genet.

[7] Lister R (2009). Human DNA methylomes at base resolution show widespread epigenomic differences. Nature.

[8] Zhou WD (2018). DNA methylation loss in late-replicating domains is linked to mitotic cell division. Nat Genet.

[9] Suzuki M (2018). Whole-genome bisulfite sequencing with improved accuracy and cost. Genome Res.

[10] Olova N (2018). Comparison of whole-genome bisulfite sequencing library preparation strategies identifies sources of biases affecting DNA methylation data. Genome Biology.

[11] Raine A, Liljedahl U, Nordlund J (2018). Data quality of whole genome bisulfite sequencing on Illumina platforms. PLoS One.

[12] Nair SS (2018). Guidelines for whole genome bisulphite sequencing of intact and FFPET DNA on the Illumina HiSeq X Ten. Epigenetics Chromatin.

[13] Libertini Emanuele, Heath Simon C, Hamoudi Rifat A, Gut Marta, Ziller Michael J, Herrero Javier, Czyz Agata, Ruotti Victor, Stunnenberg Hendrik G, Frontini Mattia, Ouwehand Willem H, Meissner Alexander, Gut Ivo G, Beck Stephan (2016). Saturation analysis for whole-genome bisulfite sequencing data. Nature Biotechnology.

[14] Libertini E (2016). Information recovery from low coverage whole-genome bisulfite sequencing. Nat Commun.

[15] NIH Roadmap Epigenomics Mapping Consortium. Standards and guidelines for whole genome shotgun bisulfite sequencing, http://www.roadmapepigenomics.org/files/protocols/data/dna-methylation/MethylC-SeqStandards_FINAL.pdf. (Accessed 16 Dec 2017).

[16] Luo C (2017). Single-cell methylomes identify neuronal subtypes and regulatory elements in mammalian cortex. Science.

[17] Khanna Anupama, Czyz Agata, Syed Fraz (2013). EpiGnome™ Methyl-Seq Kit: a novel post–bisulfite conversion library prep method for methylation analysis. Nature Methods.

[18] Parkinson NJ (2012). Preparation of high-quality next-generation sequencing libraries from picogram quantities of target DNA. Genome Res.

[19] Liu, B. J. *et al*. CpG methylation patterns of human mitochondrial DNA. *Sci Rep-Uk* **6**, 10.1038/srep23421 (2016). 10.1038/srep23421 PMC4800444 26996456

[20] Mechta, M., Ingerslev, L. R., Fabre, O., Picard, M. & Barres, R. Evidence Suggesting Absence of Mitochondrial DNA Methylation. *Front Genet* **8**, 10.3389/fgene.2017.00166 (2017). 10.3389/fgene.2017.00166 PMC5671948 29163634

[21] Schirmer Melanie, Ijaz Umer Z., D'Amore Rosalinda, Hall Neil, Sloan William T., Quince Christopher (2015). Insight into biases and sequencing errors for amplicon sequencing with the Illumina MiSeq platform. Nucleic Acids Research.

[22] Schirmer, M., D’Amore, R., Ijaz, U. Z., Hall, N. & Quince, C. Illumina error profiles: resolving fine-scale variation in metagenomic sequencing data. *Bmc Bioinformatics* **17**, 10.1186/s12859-016-0976-y (2016). 10.1186/s12859-016-0976-y PMC4787001 26968756

[23] Bibikova M (2011). High density DNA methylation array with single CpG site resolution. Genomics.

[24] Pidsley R (2016). Critical evaluation of the Illumina MethylationEPIC BeadChip microarray for whole-genome DNA methylation profiling. Genome Biol.

[25] Lehne B (2015). A coherent approach for analysis of the Illumina HumanMethylation450 BeadChip improves data quality and performance in epigenome-wide association studies. Genome Biol.

[26] Altman DG, Bland JM (1983). Measurement in Medicine - the Analysis of Method Comparison Studies. Statistician.

[27] Bland JM, Altman DG (1999). Measuring agreement in method comparison studies. Stat Methods Med Res.

[28] Cokus SJ (2008). Shotgun bisulphite sequencing of the Arabidopsis genome reveals DNA methylation patterning. Nature.

[29] QIAGEN. QIAseq Methyl Library Kit, https://www.qiagen.com/sg/shop/sequencing/qiaseq-solutions/qiaseq-methyl-library-kit/#orderinginformation. (Accessed 25 Feb 2018).

[30] Krueger Felix, Andrews Simon R. (2011). Bismark: a flexible aligner and methylation caller for Bisulfite-Seq applications. Bioinformatics.

[31] Bronner IF, Quail MA, Turner DJ, Swerdlow H (2014). Improved Protocols for Illumina Sequencing. Curr Protoc Hum Genet.

[32] Fu, Y., Wu, P. H., Beane, T., Zamore, P. D. & Weng, Z. P. Elimination of PCR duplicates in RNA-seq and small RNA-seq using unique molecular identifiers. *Bmc Genomics* **19**, 10.1186/s12864-018-4933-1 (2018). 10.1186/s12864-018-4933-1 PMC6044086 30001700

[33] Andrews KR, Good JM, Miller MR, Luikart G, Hohenlohe PA (2016). Harnessing the power of RADseq for ecological and evolutionary genomics. Nature Reviews Genetics.

[34] Hansen KD, Brenner SE, Dudoit S (2010). Biases in Illumina transcriptome sequencing caused by random hexamer priming. Nucleic Acids Res.

[35] Quail MA (2008). A large genome center’s improvements to the Illumina sequencing system. Nat Methods.

[36] Kozarewa I (2009). Amplification-free Illumina sequencing-library preparation facilitates improved mapping and assembly of (G + C)-biased genomes. Nat Methods.

[37] Nakamura K (2011). Sequence-specific error profile of Illumina sequencers. Nucleic Acids Res.

[38] Bravo HC, Irizarry RA (2010). Model-based quality assessment and base-calling for second-generation sequencing data. Biometrics.

[39] Aird D (2011). Analyzing and minimizing PCR amplification bias in Illumina sequencing libraries. Genome Biol.

[40] Benjamini Y, Speed TP (2012). Summarizing and correcting the GC content bias in high-throughput sequencing. Nucleic Acids Res.

[41] Shenker N, Flanagan JM (2012). Intragenic DNA methylation: implications of this epigenetic mechanism for cancer research. Brit J Cancer.

[42] Medvedeva Yulia A, Fridman Marina V, Oparina Nina J, Malko Dmitry B, Ermakova Ekaterina O, Kulakovskiy Ivan V, Heinzel Andreas, Makeev Vsevolod J (2010). Intergenic, gene terminal, and intragenic CpG islands in the human genome. BMC Genomics.

[43] Luo YT, Lu XM, Xie HH (2014). Dynamic Alu Methylation during Normal Development, Aging, and Tumorigenesis. Biomed Res Int.

[44] Rauscher, G. H. *et al*. Exploring DNA methylation changes in promoter, intragenic, and intergenic regions as early and late events in breast cancer formation. *Bmc Cancer* **15**, 10.1186/s12885-015-1777-9 (2015). 10.1186/s12885-015-1777-9 PMC4625569 26510686

[45] Hadfield, J. Increased read duplication on patterned flowcells- understanding the impact of Exclusion Amplification, http://core-genomics.blogspot.com/2016/05/increased-read-duplication-on-patterned.html (Accessed 18 Aug 2018).

[46] QC Fail: Sequencing. Illumina Patterned Flow Cells Generate Duplicated Sequences, https://sequencing.qcfail.com/articles/illumina-patterned-flow-cells-generate-duplicated-sequences/, (Accessed 16 Jun 2018).

[47] QC Fail: Sequencing. Illumina 2 colour chemistry can overcall high confidence G bases, https://sequencing.qcfail.com/articles/illumina-2-colour-chemistry-can-overcall-high-confidence-g-bases/, (Accessed 18 Jun 2018).

[48] Zou LS (2018). BoostMe accurately predicts DNA methylation values in whole-genome bisulfite sequencing of multiple human tissues. BMC Genomics.

[49] Chambers JC (2008). Common genetic variation near MC4R is associated with waist circumference and insulin resistance. Nat Genet.

[50] Chambers JC (2009). Common Genetic Variation Near Melatonin Receptor MTNR1B Contributes to Raised Plasma Glucose and Increased Risk of Type 2 Diabetes Among Indian Asians and European Caucasians. Diabetes.

[51] Chambers, J. C. *et al*. Epigenome-wide association of DNA methylation markers in peripheral blood from Indian Asians and Europeans with incident type 2 diabetes: a nested case-control study. *Lancet Diabetes Endo* **3**, 526–534, 10.1016/S2213-8587(15)00127-8 (2015). 10.1016/S2213-8587(15)00127-8 PMC4724884 26095709

[52] Kooner JS (2011). Genome-wide association study in individuals of South Asian ancestry identifies six new type 2 diabetes susceptibility loci. Nat Genet.

[53] illumina. bcl2fastq2 Conversion Software v2.20, http://sapac.support.illumina.com/downloads/bcl2fastq-conversion-software-v2-20.html?langsel=/sg/. (Accessed 1 Feb 2018).

[54] Babraham Bioinformatics. FastQC, https://www.bioinformatics.babraham.ac.uk/projects/fastqc/. (Accessed 20 Dec 2017).

[55] Babraham Bioinformatics. Trim Galore, https://www.bioinformatics.babraham.ac.uk/projects/trim_galore/. (Accessed 21 Dec 2017).

[56] Martin M (2011). Cutadapt removes adapter sequences from high-throughput sequencing reads. EMBnet.journal.

[57] Krueger, F. Bismark Bisulfite Mapper, https://github.com/FelixKrueger/Bismark/tree/master/Docs. (Accessed 1 Dec 2017).

[58] Swift Biosciences, l. Accel-NGS®1S plus & Methyl-Seq: tail trimming for better data, https://swiftbiosci.com/wp-content/uploads/2016/09/16-0853-Tail-Trim-TN.pdf. (Accessed 18 Dec 2017).

[59] Li H (2009). The Sequence Alignment/Map format and SAMtools. Bioinformatics.

[60] Okonechnikov K, Conesa A, Garcia-Alcalde F (2016). Qualimap 2: advanced multi-sample quality control for high-throughput sequencing data. Bioinformatics.

[61] broad institute. Picard, http://broadinstitute.github.io/picard/. (Accessed 18 Jan 2018).

[62] Karolchik D (2018). The UCSC Table Browser data retrieval tool. Nucleic Acids Res.

[63] Wen L (2014). Whole-genome analysis of 5-hydroxymethylcytosine and 5-methylcytosine at base resolution in the human brain. Genome Biol.

