# Supplementary material for: Systematic evaluation of library preparation methods and sequencing platforms for high-throughput whole genome bisulfite sequencing
Source: Sci Rep. 2019 Jul 17;9:10383. doi: 10.1038/s41598-019-46875-5 (PMC6637168; doi:10.1038/s41598-019-46875-5)

Systematic evaluation of library preparation methods and sequencing platforms for high-throughput whole genome bisulfite sequencing

Li Zhou[1], Hong Kiat Ng[1], Daniela I. Drautz-Moses[2], Stephan C. Schuster[2], Stephan Beck[3], Changhoon Kim[4], John Campbell Chambers[1,5,6,7], Marie Loh[1,5,8*]

[1]Lee Kong Chian School of Medicine, Nanyang Technological University, Singapore 308232

[2]Singapore Centre for Environmental Life Sciences Engineering, Nanyang Technological University, Singapore 637551

[3]University College London Cancer Institute, 72 Huntley Street, London, WC1E 6BT, UK

[4]MACROGEN, Inc., Seoul 08511, Republic of Korea

[5]Department of Epidemiology and Biostatistics, Imperial College London, London W2 1PG, UK

[6]Department of Cardiology, Ealing Hospital, London North West Healthcare NHS Trust, Southall UB1 3HW, UK

[7]Imperial College Healthcare NHS Trust, London W12 0HS, UK

[8]Translational Laboratory in Genetic Medicine, Agency for Science, Technology and Research, Singapore (A*STAR), 8A Biomedical Grove, Immunos, Level 5, Singapore 138648

Li Zhou: zhou.li@ntu.edu.sg

Hong Kiat Ng: hongkiat.ng@ntu.edu.sg

Daniela I. Drautz-Moses: danielamoses.scelse@gmail.com

Stephan C. Schuster: stephan.c.schuster@gmail.com

Stephan Beck: s.beck@ucl.ac.uk

Changhoon Kim: kimchan@macrogen.com

John Campbell Chambers: john.chambers@ntu.edu.sg

*Marie Loh: marie_loh@ntu.edu.sg. Corresponding author.

Supplementary Figure Legends

Supplementary Fig. 1: Comparison of quality metric across library preparation methods

a) Raw reads from Read 1 and Read 2 with sequencing quality >Q20 or >Q30. **b)** Bases trimmed due to low quality (<Q20) for Read 1 and Read 2. **C)** Bases containing adaptor sequences for Read 1 and Read 2. All libraries were sequenced on the HiSeq X platform. n.s.: not statistically significant, P>0.05, *: P<0.05, **: P<0.01, ***: P<0.001. Error bars represent standard error of mean.

Supplementary Fig. 2: Outline of workflow for data quality analysis and processing pipeline

Sequence quality was first checked by FastQC v0.11.5 [1]. Adaptors and low quality sequences (Phred<20) were trimmed by Trim Galore v0.4.4_dev and cutadapt v1.15 [2,3]. Trimmed reads were checked by FastQC again before mapping to bisulfite-converted hg19 genome by bismark [4]. Mapped reads were deduplicated by deduplicate_bismark and sorted by samtools v1.3 for further analysis [5]. Depth of coverage distribution per chromosome was subsequently generated by Qualimap v2.2.1 [6], with read depth of coverage and insert size analyzed by Picard tools v2.18.16 [7]. DNA methylated sites were identified, extracted and counted by bismark_methylation_extractor.

Supplementary Fig. 3: Scatter plots comparing methylation levels measured by WGBS and array for all samples.

Pearson correlation coefficient (r) between methylation levels measured by WGBS and array are shown in each panel for each sample. The analysis is restricted to only CpG sites measured by both WGBS (Swift method on HiSeq X platform) and array, with a minimal depth of 10x in WGBS.

Supplementary Fig. 4: Bland-Altman plots comparing methylation levels reported by the methylation arrays and WGBS.

CpGs are stratified by different depth levels, with only CpG sites measured by both WGBS (Swift method on HiSeq X platform) and array in each depth bin. Only one representative sample is shown here (Sample 1).

Supplementary Fig. 5: Comparison of performance between WGBS libraries generated by Swift, TruSeq and QIAseq library preparation kits versus 450K and EPIC methylation arrays

a) CpG site coverage. **b)** CpG site coverage at different genomic features. The percentages were calculated by dividing the number of CpG sites covered with a minimum depth of 10x for each genomic feature by the total number of CpG sites in the genome for the corresponding genomic feature. Inset: Distribution of CpG sites in the genome by genomic features. Bars show average values, with error bars representing standard error of mean. **c)** Bland-Altman plots comparing methylation levels reported by WGBS and the methylation arrays. Upper (all probe types): WGBS versus 450K (left), WGBS versus EPIC (right). Lower (stratified by Type I and Type II

probes): WGBS versus 450K (left), WGBS versus EPIC (right). Each line represents one sample (library). **d)** Comparison of standard deviation (SD) of methylation levels of replicate samples between WGBS and methylation arrays. QIAseq kit was not included in the current analysis as replicates were not performed in view of its inferior performance as concluded from earlier sections. CpG sites were binned according to their average coverage, inclusive of the lower limit and exclusive of the upper limit. For WGBS data, only CpG sites with a minimal depth of 10x were used across all analyses shown here. For **c** and **d**, only CpG sites found in both WGBS and array data are considered in the analyses. All WGBS data included in this analysis were generated on the HiSeq X platform.

Supplementary Fig. 6: Comparison of performance between WGBS libraries generated by Swift preparation kit on the NovaSeq platform versus 450K and EPIC methylation arrays

a) CpG site coverage. **b)** CpG site coverage at different genomic features. The percentages were obtained by dividing the number of CpG sites covered with a minimum depth of 10x for each genomic feature by the total number of CpG sites in the genome for the corresponding genomic feature, multiplied by 100%. Inset: Distribution of CpG sites in the genome by genomic features. Bars show average values, with error bars representing standard error of mean. **c)** Bland-Altman plots comparing methylation levels reported by WGBS and the methylation arrays. Upper (all probe types): WGBS versus 450K (left), WGBS versus EPIC (right). Lower (stratified by Type I and Type II probes): WGBS versus 450K (left), WGBS versus EPIC (right). Each line represents one sample (library). **d)** Comparison of standard deviation (SD) of methylation levels of replicate samples between WGBS and

methylation arrays. TruSeq and QIAseq methods were excluded from this analysis kits as the focus here was on the best performing WGBS library preparation method (i.e. Swift) as determined in earlier sections. CpG sites were binned according to their average coverage, inclusive of the lower limit and exclusive of the upper limit. For WGBS data, only CpG sites with a minimal depth of 10x were used across all analyses shown here. For **c** and **d**, only CpG sites found in both WGBS and array data are considered in the analyses.

Supplementary Table Legends

Supplementary Table 1: P-values for testing of nucleotide amplification biases at each category for libraries generated by Swift, TruSeq and QIAseq library preparation kits

A) One-sample t-test was performed across all categories (nucleotide/dinucleotide) for each library preparation method (tested against expected mean of zero in the case of no bias). B) Paired-sample t-test was performed across all categories (nucleotide/dinucleotide) for each library preparation method to test for differences between providers. Bonferroni correction was performed to account for the multiple testing across all categories. All libraries included in this analysis were sequenced on the HiSeq X platform.

Supplementary Table 2: Genome coverage at each minimum depth for libraries generated by Swift, TruSeq and QIAseq library preparation methods

A) Genome coverage at respective minimum depths across library preparation methods B) P-values from Tukey HSD performed for pairwise comparisons between library preparation methods at each minimum sequencing depth (following ANOVA test across all three categories). Bonferroni correction was performed to account for the multiple testing across all depths. All libraries included in this analysis were sequenced on the HiSeq X platform.

Supplementary Table 3: CpG site coverage covered at each minimum depth for libraries generated by Swift, TruSeq and QIAseq library preparation kits and corresponding P-values for testing of no difference in coverage between library preparation methods

A) CpG site coverage at respective minimum depths across library preparation methods B) P-values from Tukey HSD performed for pairwise comparisons between library preparation methods at each minimum sequencing depth (following ANOVA test across all three categories). Bonferroni correction was performed to account for the multiple testing across all depths. All libraries included in this analysis were sequenced on the HiSeq X platform.

Supplementary Table 4: Methylation levels observed in mitochondria for libraries generated by Swift, TruSeq and QIAseq library preparation methods and corresponding P-values for testing against expectation of no methylation

One-sample t-test was performed for methylation levels observed in mitochondria against expected mean of zero (in the case of no methylation) for each sample, organized by provider and library preparation methods. All libraries included in this analysis were sequenced on the HiSeq X platform.

Supplementary Table 5: Genome coverage at each minimum depth for libraries generated by Swift, TruSeq and QIAseq library preparation methods across different number of raw read pairs

Results shown here are obtained from downsampling analyses for minimum depths ranging from 1-100x for library sizes consisting of 100-1000M raw read pairs. All available samples were pooled for the downsampling analyses. All libraries included in this analysis were sequenced on the HiSeq X platform.

Supplementary Table 6: CpG site coverage at each minimum depth for libraries generated by Swift, TruSeq and QIAseq library preparation methods across different number of raw read pairs

Results shown here are obtained from downsampling analyses for minimum depths ranging from 1-100x for library sizes consisting of 100-1000M raw read pairs. All available samples were pooled for the downsampling analyses. All libraries included in this analysis were sequenced on the HiSeq X platform.

Supplementary Table 7: CpG site coverage for libraries sequenced on NovaSeq and HiSeq X at each normalized minimum depth and corresponding P-values for testing of no difference in coverage between the two platforms

A) CpG site coverage at respective minimum depths across sequencing platforms B) P-values from paired sample t-tests performed for pairwise comparisons between sequencing platforms at each minimum sequencing depth. Bonferroni correction was performed to account for the multiple testing across all depths. All libraries included in this analysis were prepared by the Swift library preparation method.

Supplementary Table 8: P-values for testing of nucleotide amplification biases at each category for libraries sequenced on NovaSeq and HiSeq X

Paired-sample t-test was performed across all categories (nucleotide/dinucleotide) to test for differences in nucleotide amplification biases. Nucleotide amplification biases were expressed as the logarithm 2 transformed ratio of observed to expected coverage for different nucleotide and dinucleotide combinations. Bonferroni correction was performed to account for the multiple testing across all categories. All libraries included in this analysis were prepared by the Swift library preparation method.

Supplementary Table 9: Genome coverage at each minimum depth for libraries sequenced on NovaSeq and HiSeq X across different number of raw read pairs

Results shown here are obtained from downsampling analyses for minimum depths ranging from 1-200x for library sizes consisting of 100-4000M raw read pairs. All available samples were pooled for the downsampling analyses. All libraries included in this analysis were prepared by the Swift library preparation method.

Supplementary Table 10: CpG site coverage at each minimum depth for libraries sequenced on NovaSeq and HiSeq X across different number of raw read pairs

Results shown here are obtained from downsampling analyses for minimum depths ranging from 1-200x for library sizes consisting of 100-4000M raw read pairs. All available samples were pooled for the downsampling analyses. All libraries included in this analysis were prepared by the Swift library preparation method.

Supplementary Table 11: Standard deviation at each depth bin and methylation level bin for A) WGBS (Swift and TruSeq library preparation methods) and B) methylation array (450K)

Standard deviation (SD) of methylation levels of replicate samples between WGBS and methylation arrays. CpG sites were binned according to their average coverage, inclusive of the lower limit and exclusive of the upper limit. Methylation bin size was set at 1%. For WGBS data, only CpG sites with a minimal depth of 10x were used across all analyses shown here. Only CpG sites found in both WGBS and array data are considered in the analyses. All WGBS data included in this analysis were generated on the HiSeq X platform. QIAseq kit was not included in the current analysis as replicates were not performed in view of its inferior performance as concluded from earlier sections.

Supplementary Table 12: Sample size calculation to detect differences in methylation levels between cases and controls via WGBS

Sample size was calculated for a case-control setting (1:1 case-control ratio) at 5.00E-8% significance level and 80% power to detect differences in methylation levels between 1-4%. Total SD was calculated as the square root of the sum of population variance (SD was set at 4%; estimated from previous studies) and WGBS variance.

References

1 Babraham Bioinformatics. FastQC. https://www.bioinformatics.babraham.ac.uk/projects/fastqc/. (Accessed 20 Dec 2017).

2 Babraham Bioinformatics. Trim Galore. https://www.bioinformatics.babraham.ac.uk/projects/trim_galore/. (Accessed 21 Dec 2017).

3 Martin, M. Cutadapt removes adapter sequences from high-throughput sequencing reads. *EMBnet.journal* **17**, 10-12, doi:http://dx.doi.org/10.14806/ej.17.1.200 (2011).

4 Krueger, F. & Andrews, S. R. Bismark: a flexible aligner and methylation caller for Bisulfite-Seq applications. *Bioinformatics* **27**, 1571-1572, doi:10.1093/bioinformatics/btr167 (Accessed 1 Dec 2017).

5 Li, H. *et al.* The Sequence Alignment/Map format and SAMtools. *Bioinformatics* **25**, 2078-2079, doi:10.1093/bioinformatics/btp352 (2009).

6 Okonechnikov, K., Conesa, A. & Garcia-Alcalde, F. Qualimap 2: advanced multi-sample quality control for high-throughput sequencing data. *Bioinformatics* **32**, 292-294, doi:10.1093/bioinformatics/btv566 (2016).

7 broad institute. Picard. http://broadinstitute.github.io/picard/. (Accessed 18 Jan 2018).

Supplementary Fig. 1

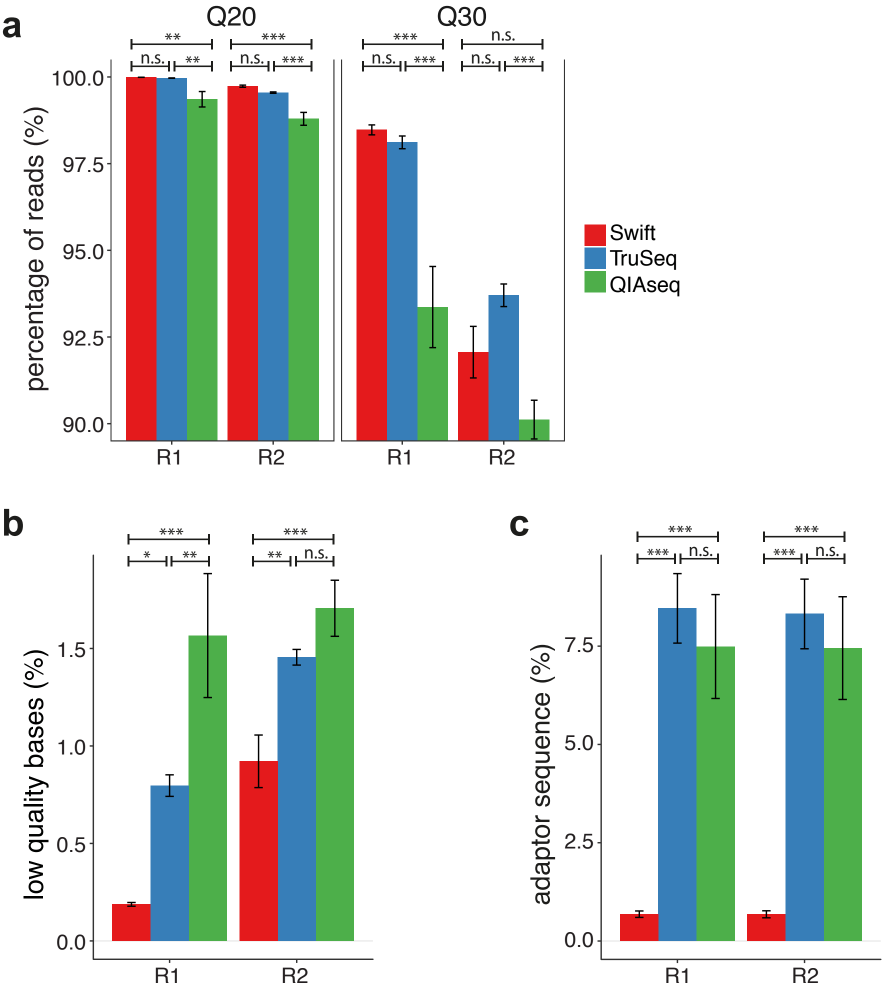

Supplementary Fig. 2

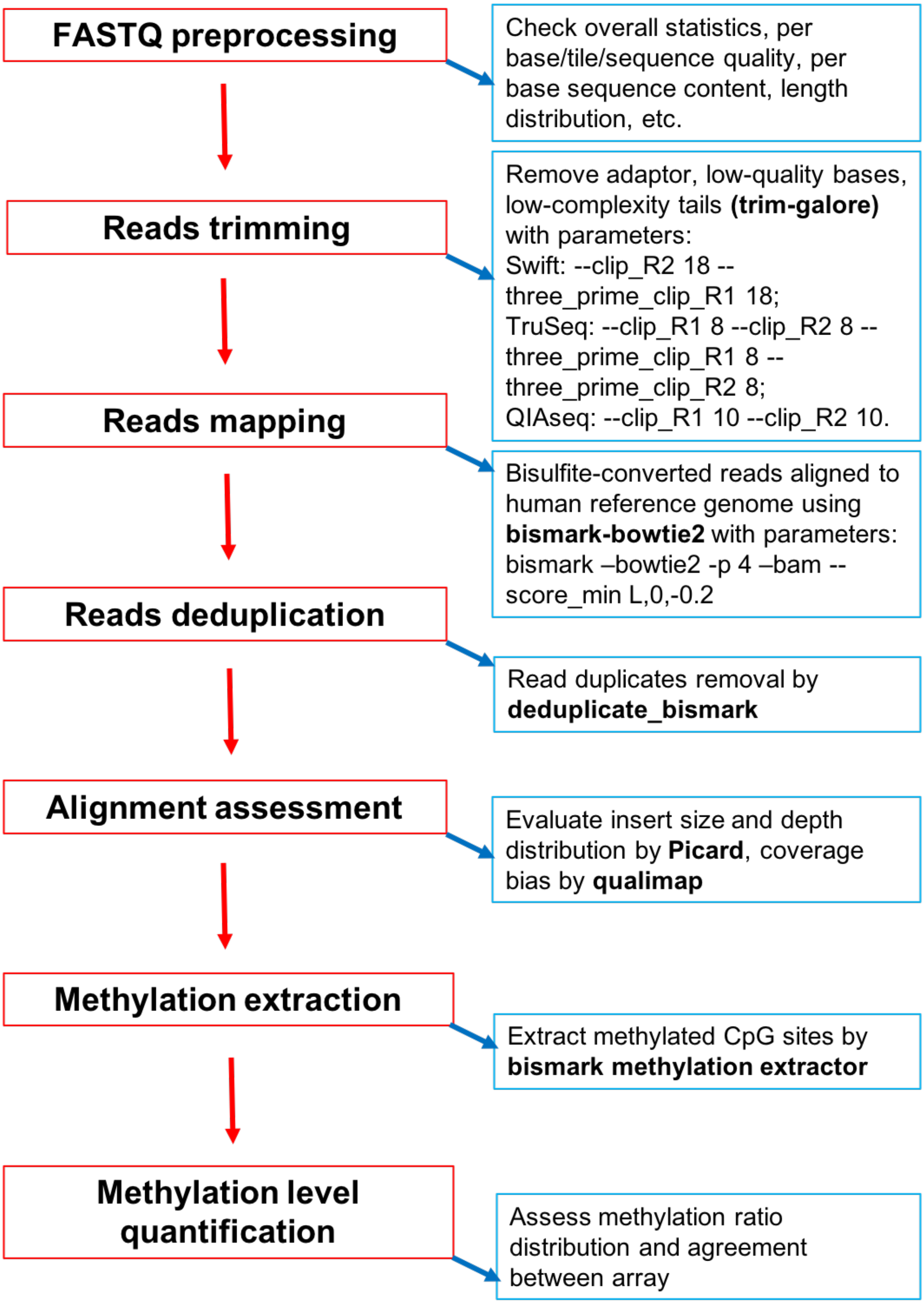

Supplementary Fig. 3

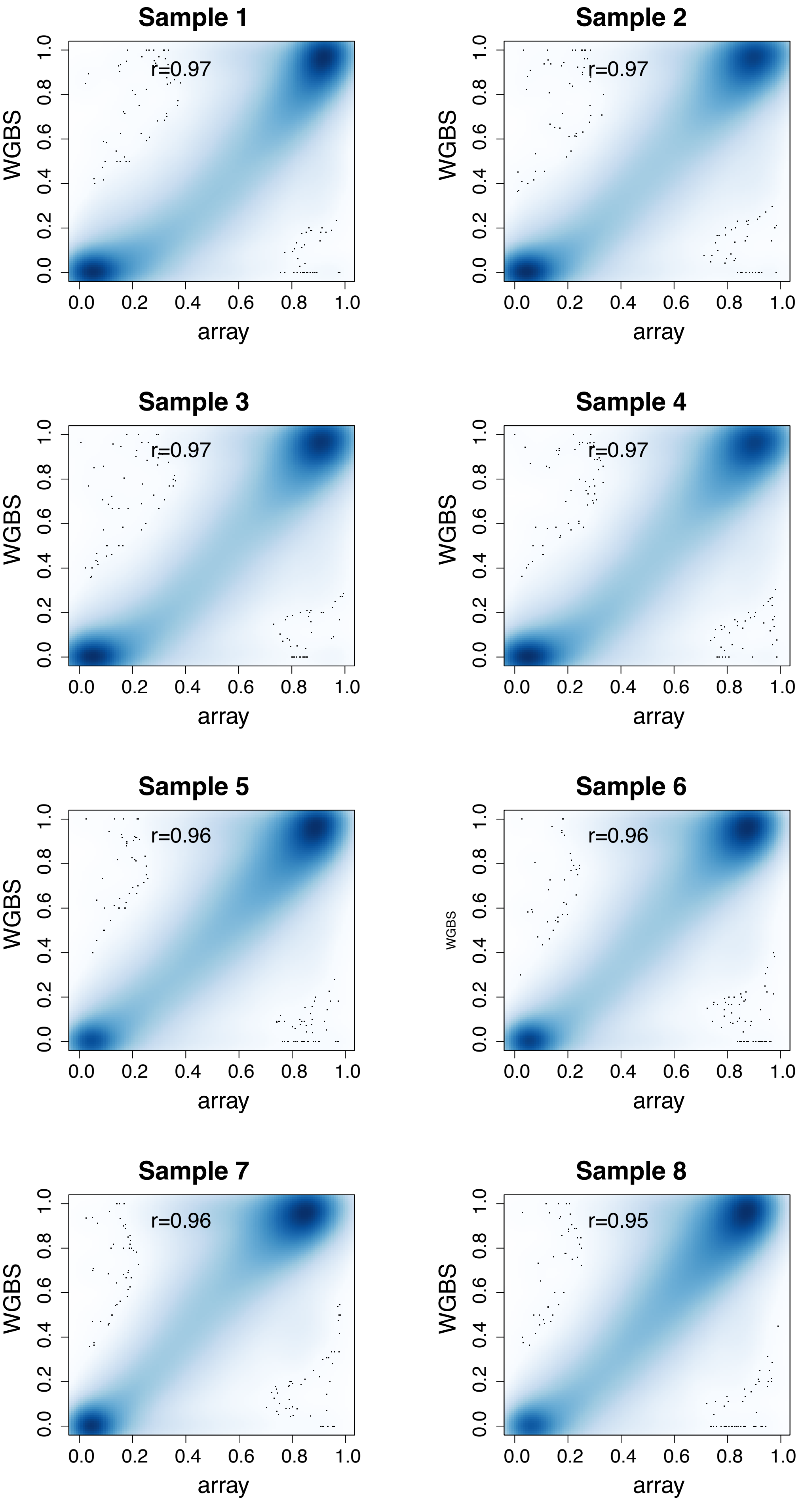

Supplementary Fig. 4

a

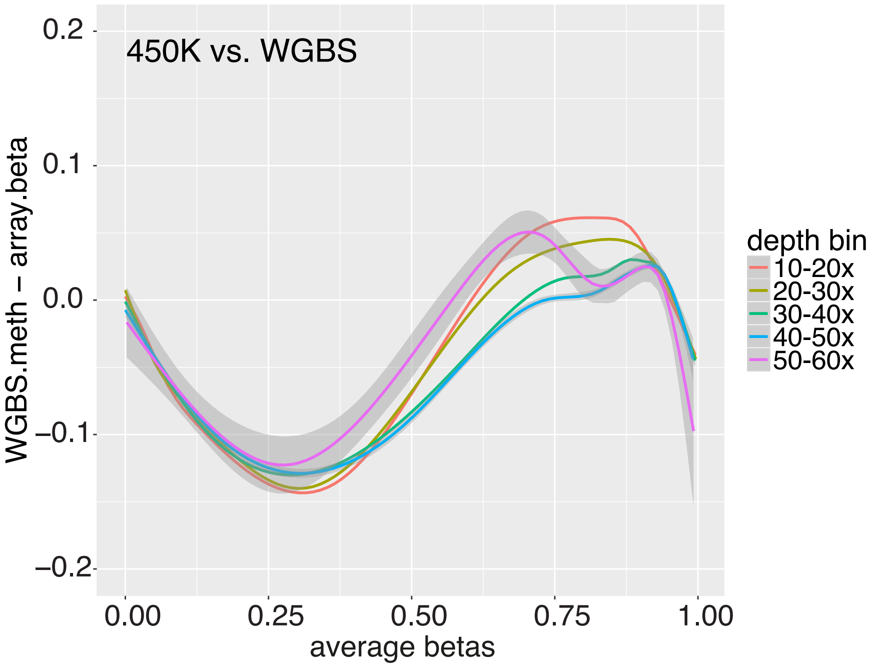

b

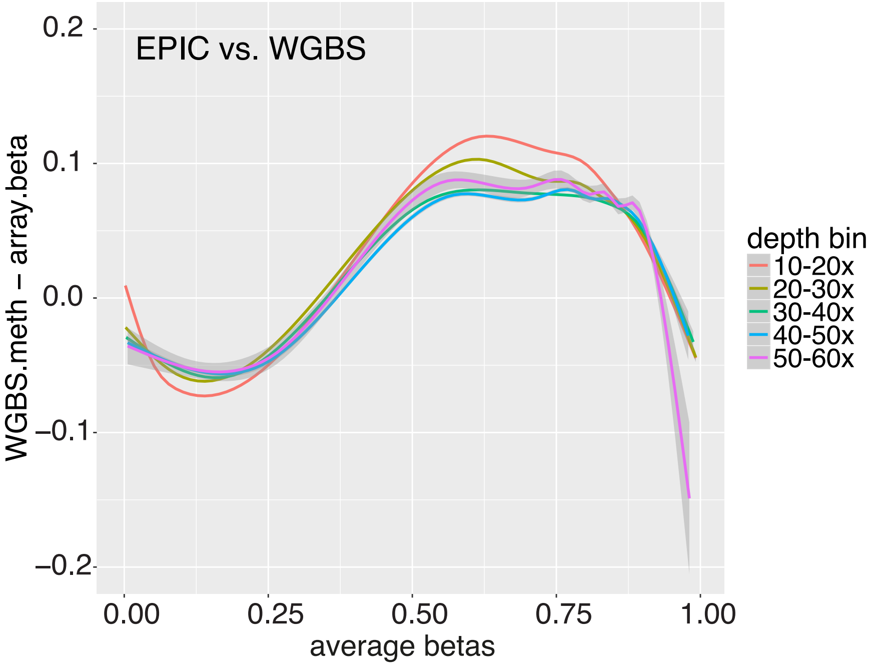

Supplementary Fig. 5

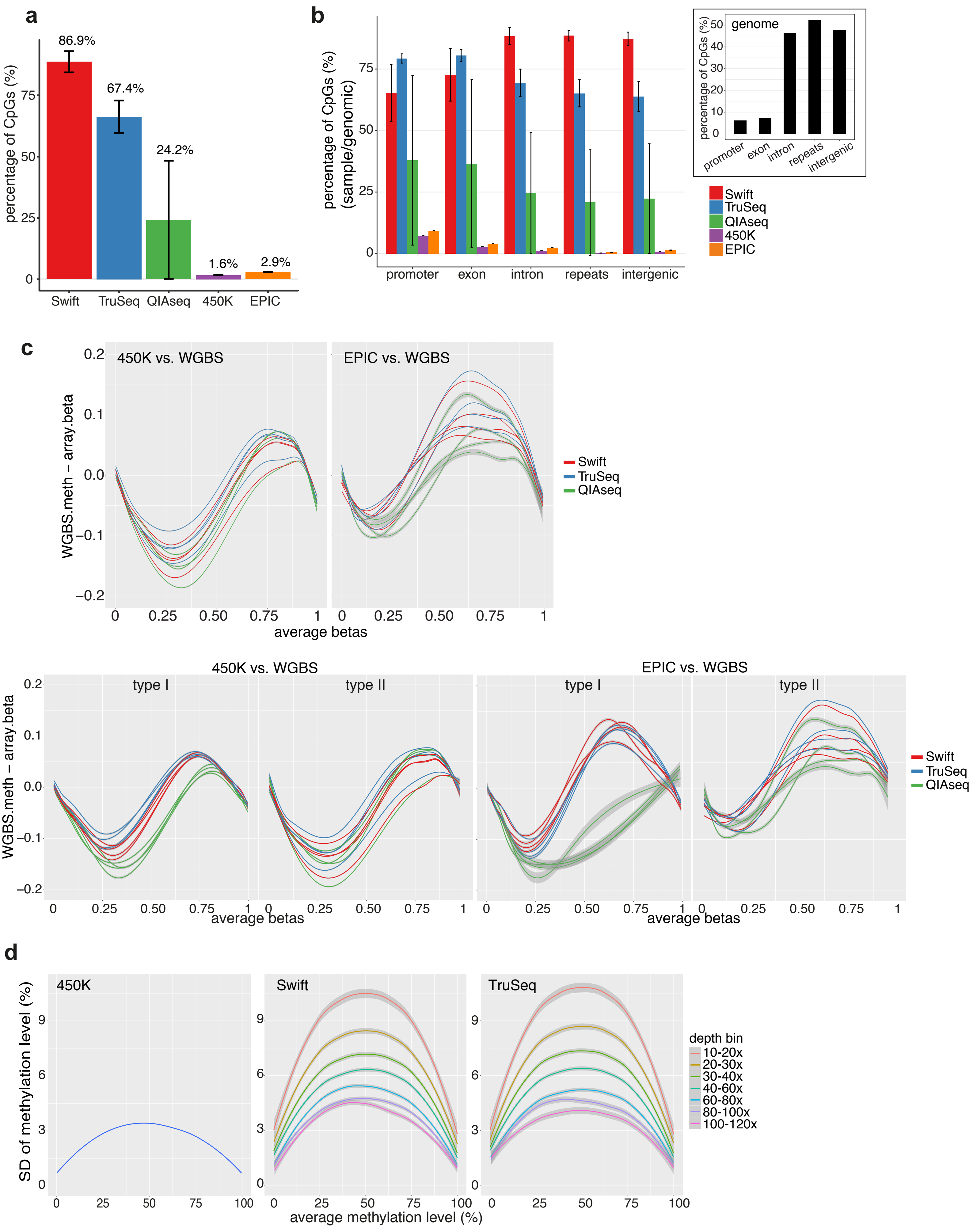

Supplementary Fig. 6

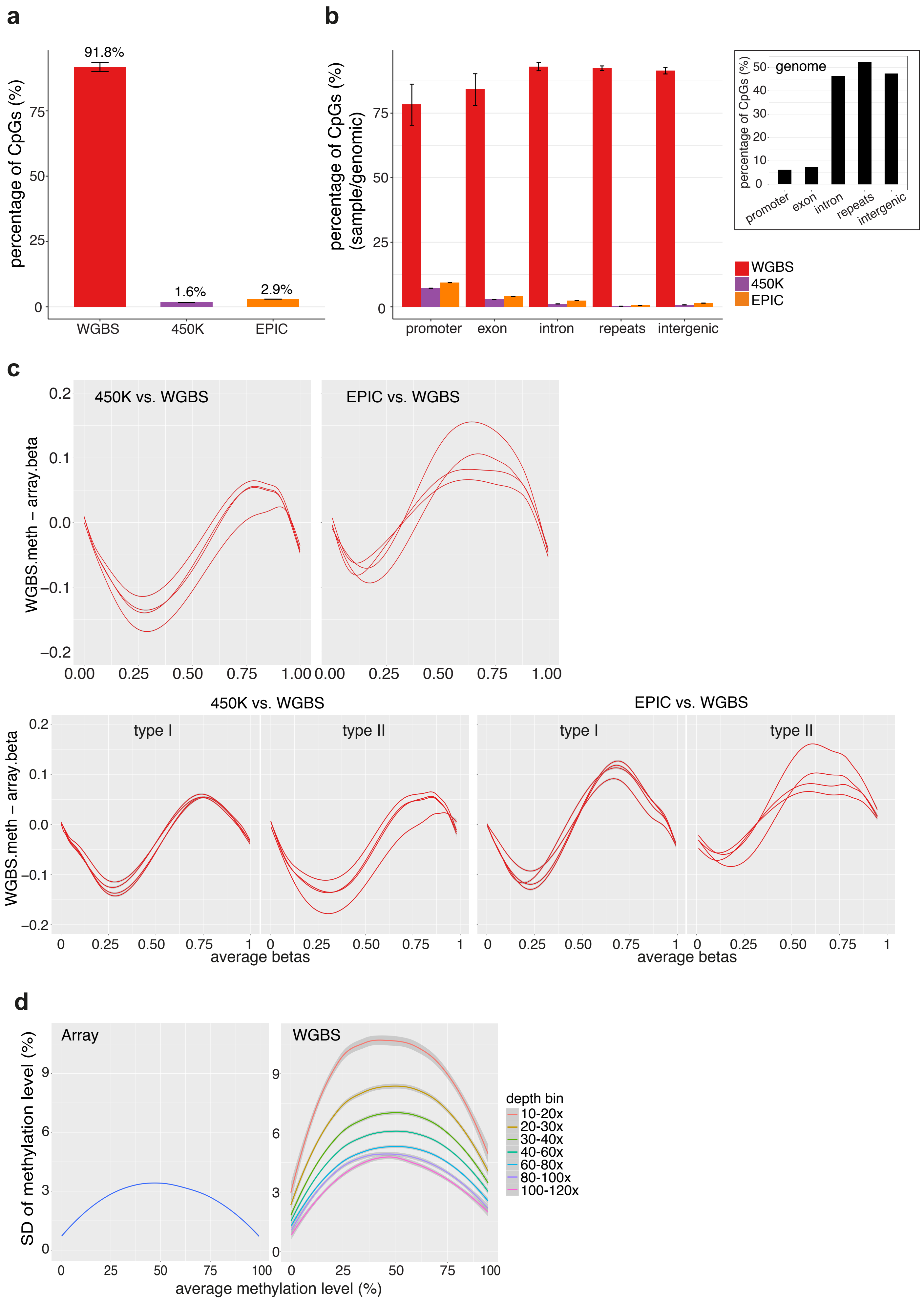

Supplement: Supplementary file 2 — Supplementary Information [file 41598_2019_46875_MOESM2_ESM.pdf]
